# Host and microbiome features of secondary infections in lethal covid-19

**DOI:** 10.1016/j.isci.2022.104926

**Published:** 2022-08-13

**Authors:** Martin Zacharias, Karl Kashofer, Philipp Wurm, Peter Regitnig, Moritz Schütte, Margit Neger, Sandra Ehmann, Leigh M. Marsh, Grazyna Kwapiszewska, Martina Loibner, Anna Birnhuber, Eva Leitner, Andrea Thüringer, Elke Winter, Stefan Sauer, Marion J. Pollheimer, Fotini R. Vagena, Carolin Lackner, Barbara Jelusic, Lesley Ogilvie, Marija Durdevic, Bernd Timmermann, Hans Lehrach, Kurt Zatloukal, Gregor Gorkiewicz

**Affiliations:** 1 Diagnostic and Research Institute of Pathology, Medical University of Graz, Neue Stiftingtalstrasse 6, 8010 Graz, Austria; 2 Alacris Theranostics GmbH, Max-Planck-Strasse 3, 12489 Berlin, Germany; 3 Ludwig Boltzmann Institute for Lung Vascular Research, Neue Stiftingtalstrasse 6/VI, 8010 Graz, Austria; 4 Diagnostic and Research Institute of Hygiene, Microbiology and Environmental Medicine, Medical University of Graz, Neue Stiftingtalstrasse 6, 8010 Graz, Austria; 5 Max Planck Institute for Molecular Genetics, Ihnestrasse 63, 14195 Berlin, Germany

**Keywords:** Immunology, Virology, Microbiome

## Abstract

Secondary infections contribute significantly to covid-19 mortality but driving factors remain poorly understood. Autopsies of 20 covid-19 cases and 14 controls from the first pandemic wave complemented with microbial cultivation and RNA-seq from lung tissues enabled description of major organ pathologies and specification of secondary infections. Lethal covid-19 segregated into two main death causes with either dominant diffuse alveolar damage (DAD) or secondary pneumonias. The lung microbiome in covid-19 showed a reduced biodiversity and increased prototypical bacterial and fungal pathogens in cases of secondary pneumonias. RNA-seq distinctly mirrored death causes and stratified DAD cases into subgroups with differing cellular compositions identifying myeloid cells, macrophages and complement C1q as strong separating factors suggesting a pathophysiological link. Together with a prominent induction of inhibitory immune-checkpoints our study highlights profound alterations of the lung immunity in covid-19 wherein a reduced antimicrobial defense likely drives development of secondary infections on top of SARS-CoV-2 infection.

## Highlights

• Covid-19 autopsy cohort complemented with microbial cultivation and deep sequencing
• Major death causes stratify into DAD and secondary pneumonias
• Prototypical bacterial and fungal agents are found in secondary pneumonias
• Macrophages and C1q stratify DAD subgroups and indicate immune impairment in lungs

## Graphical abstract

**Figure:**
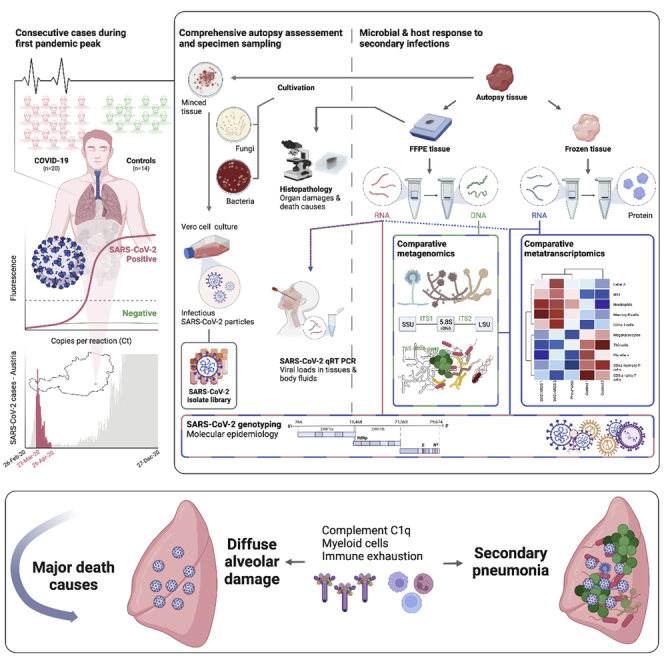

## Introduction

Covid-19 originates from infection of the upper respiratory tract with SARS-CoV-2, which can progress into severe acute lung injury (ALI). Based on the tissue-typic expression of the viral host-entry receptor ACE2 and certain proteases (e.g. TMPRSS2) facilitating cellular uptake, also other organs like the kidney could be directly infected (Hou et al., 2020b). In addition, severe disturbances of immune and coagulation systems during covid-19 lead to a multifaceted disease with variable multi-organ damages (Ramlall et al., 2020). A consistent finding in severe covid-19 is initial immune hyperactivation (called “cytokine storm”) leading to subsequent immune exhaustion, a phenomenon also known in other severe infections (Blanco-Melo et al., 2020; Lowery et al., 2021; Roquilly et al., 2020; Wang et al., 2021). Consequently, secondary infections which develop on top of SARS-CoV-2 infection contribute significantly to covid-19 mortality similar to severe influenza (Buehler et al., 2021). Curiously, the pathophysiology leading to the development of secondary lung infections is generally poorly understood. We performed an autopsy study of 20 consecutive covid-19 patients who died during the first pandemic wave. Full autopsies were performed and various specimen types were collected for tissue-based investigations, molecular measures including deep sequencing and cultivation of virus and other microbes. Integrating all information gained from this “holistic” autopsy approach allowed us to gain a deeper understanding of host and microbial factors contributing to secondary infections as a major sequel of lethal covid-19.

## Results

### Autopsy cohort, SARS-CoV-2 body distribution and genotyping

**Figure 1:**
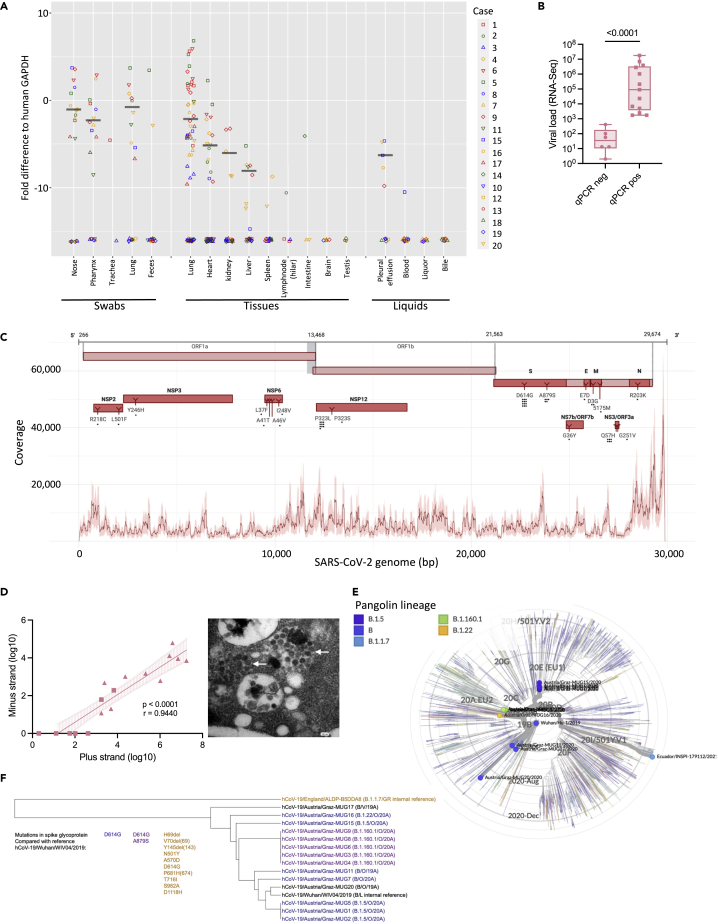
SARS-CoV-2 tissue distributions, genotyping and virus cultivation (A) SARS-CoV-2 loads (compared to human glyceraldehyde 3-phosphate dehydrogenase, GAPDH) and tissue distributions derived from postmortem sampling (median highlighted). Case numbers are given on the right. (B) Significant association of qRT-PCR positivity (n-gene) with viral loads determined by RNAseq of lung tissues (Mann-Whitney test). (C) Distribution of viral reads generated from lung tissues along the SARS-CoV-2 genome. Cumulative coverage of plus and minus strand transcripts is shown (median in bold). Identified nucleotide and amino-acid changes in comparison to the Wuhan reference strain are indicated. (D) Correlation of SARS-CoV-2 plus and minus strand reads with cultivation (Spearman correlation). Triangles specify cultivation-positive samples. EM picture showing viral particles in Vero CCL-81 cells (arrows). (E) Cladogram showing detected virus genotypes within a global context. The Wuhan reference strain (center) and the UK variant B.1.1.7 (Ecuador/INSPI-179112/2021) are included for comparisons. The pangolin lineage designation is used to specify viral genotypes. (F) Dendrogram showing detected viral genotypes. Corresponding mutations in the S protein are indicated and virus strains are color coded accordingly.

Twenty consecutive covid-19 patients were examined post-mortem (Figure S1). Thirteen cases were males and 7 were females; their ages ranged from 53 to 93 years (median: 79 years). All had multiple comorbidities typically prevalent in severe covid-19. In addition, 14 age-matched non-covid-19 controls who died within the same time period were included for comparisons (Tables S1 and S2). Patients were tested for SARS-CoV-2 tissue distributions by quantitative RT-PCR (target: nucleocapsid-gene) and most positive samples with the highest viral loads originated from the respiratory tract, followed by myocardium, liver, kidney and pleural effusions. Other tissues and body liquids were positive only in single cases or tested overall negative (Figure 1A). Notably, deep RNA-seq generated from lung tissues revealed SARS-CoV-2 transcripts in each covid-19 case, including the four qRT-PCR negative ones, showing increased sensitivity of deep transcriptomic analysis (127 ± 29 million reads were generated per sample on average; Figure 1B). The viral genome was entirely captured by RNA-seq yielding more plus-strand reads (mean: 37.89 reads per million; range: 0.02–131,165.41) than minus-strand reads (mean: 1.81 reads per million; range: 0–484.81; Figure 1C). In addition, 11 SARS-CoV-2 strains could be cultivated from post-mortem lung tissues using Vero cells (Table S3). Successful virus cultivation significantly correlated with abundance of SARS-CoV-2 reads (Figures 1D and S2).

SARS-CoV-2 genotyping facilitated by PCR and sequencing directly from autopsy specimens yielded 14 complete viral genomes (Table S4). Nine different sequence variants were detected showing up to 12 nucleotide changes compared to the reference (SARS-CoV-2 Wuhan-Hu-1; total genome size 29,903 bp; Table S5). Strains corresponded to the pangolin lineages B.1.22, B1.5, B and B.1.160.1, respectively (clades 19A and 20A), representing the dominant genotypes of the first pandemic wave (Figure 1E). Twelve strains harbored a D614G mutation in the spike (S) protein, which leads to increased viral transmissibility and, therefore, this genotype superseded the wild-type strain already early in the pandemic (Hou et al., 2020a). We identified also 2 viral clusters in our cohort, cluster 1 (case 3, 4, 6, 8, and 9) and cluster 2 (case 1, 2, and 5), respectively (Figure 1F). Notably, cases 6, 8 and 9 from cluster 1 originated from the same residential care home and all cases from cluster 2 stayed in the same hospital ward before covid-19. Thus, it is very likely that these individuals were infected from the same sources and/or transmission occurred.

### Major organ pathologies and death causes

**Figure 2:**
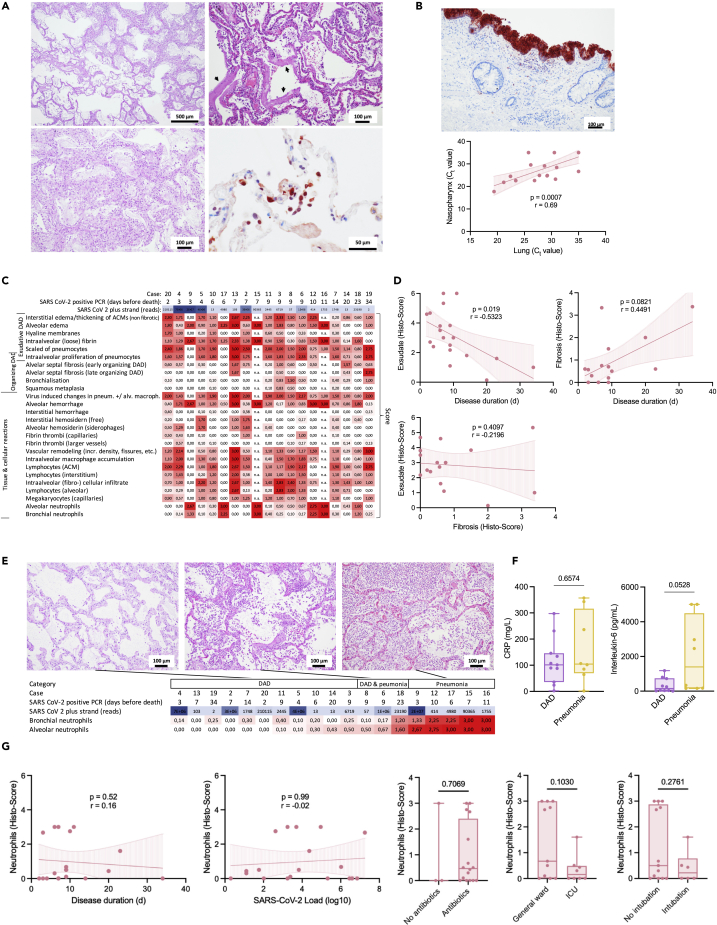
Lung pathology of lethal covid-19 stratifies into DAD and pneumonia (A) Histological representation of DAD in lungs. A patchy representation of DAD is shown (left). Hyaline membranes (arrows) as a hallmark lesion of early DAD (top right). Immunohistochemical detection (nucleoprotein antibody) of SARS-CoV-2 infected pneumocytes (bottom right). (B) (Top) Immunohistochemical detection of SARS-CoV-2 infected respiratory epithelium of the nasopharynx. (Bottom) Correlation between SARS-CoV-2 loads in the nasopharyngeal mucosa and lung tissue determined by qRT-PCR (Spearman correlation). (C) Scoring of prevalent histopathology patterns in lungs. Cases are ordered according to duration of disease. (D) Correlation analysis of early and late DAD histopathology features and disease duration (Spearman r). (E) Main discrimination of lung pathology according to DAD and pneumonia patterns. Cases are ordered according to alveolar neutrophil scores. (F) Serum C-reactive protein (CRP) and interleukin-6 (IL-6) levels in DAD and pneumonia cases (Mann-Whitney test). (G) Correlation analyses of neutrophil abundance and clinical parameters (Spearman r; Mann-Whitney test).

Lungs showed the dominant pathologies in relation to covid-19, only one case (#1) presented with acute myocardial infarction as the ascribed death cause. Diffuse alveolar damage (DAD), the histopathological representation of ALI, in a patchy distribution and often prevalent in multiple lung segments was the major finding in 11 cases. Early exudative stages and later organizing stages of DAD were found within the same patient together, often adjacent to nearly normal or less affected parenchyma indicating ongoing tissue damage (Figures 2A and S3–S5). Also, a significant positive correlation of SARS-CoV-2 loads from nasopharyngeal tissues to lungs was found (Figure 2B) likely suggesting active seeding of infectious particles from the upper respiratory tract via micro-aspiration (Hou et al., 2020b). We extensively assessed microscopic lung features (see STAR Methods for details of histopathological scoring) to specify and grade the severity of lesions and also to capture the heterogeneity of different lung pathologies. Features greatly varied between cases and the majority of patterns did not correlate with disease duration (defined as the interval between the first SARS-CoV-2 positive PCR and death) or viral loads (Figure 2C). Although early DAD features (intra-alveolar edema, hyaline membranes) correlated positively with shorter disease duration and late features (fibrosis) increased with disease duration, early and late features were often intermixed showing no inverse correlation (Figure 2D) corroborating findings also by other studies (Borczuk et al., 2020). Of importance, the clearest discriminating feature of cases was the presence of neutrophilic granulocytes, indicative of secondary infections (i.e., “pneumonia”), in comparison to DAD. Three cases showed DAD superimposed with acute inflammation and 5 cases showed mainly pneumonia as the dominant pathology, wherein DAD was only focally visible overlaid with dense inflammation. Altogether, 16 cases showed neutrophilic granulocytes present in bronchi, bronchioli, or alveoli suggestive of secondary infections. Thus, lung histopathology in lethal covid-19 could be stratified into DAD, DAD superimposed with pneumonia and dominating pneumonia (Figures 2E and S3). Pneumonia cases showed increased IL-6 levels compared with pure DAD cases, whereas CRP levels were weaker discriminators (Figure 2F). It is noteworthy that neither disease duration nor viral loads correlated with the presence of neutrophils, nor did any other clinical parameter (Figures 2G and S6). Other organs showed features of preexisting comorbidities including arteriosclerosis, hypertension and diabetes, especially in the kidneys, wherein SARS-CoV-2 could be detected in tubular epithelia by positive immunohistochemistry (Figure S7). Heart and liver specimens revealed no clear evidence of direct SARS-CoV-2 carriage or features of myocarditis or hepatitis (Figures S8 and S9). A detailed summary of organ histopathologies is given in the supplementary material (Table S6).

### Lung microbiome alterations and secondary infections in lethal covid-19

**Figure 3:**
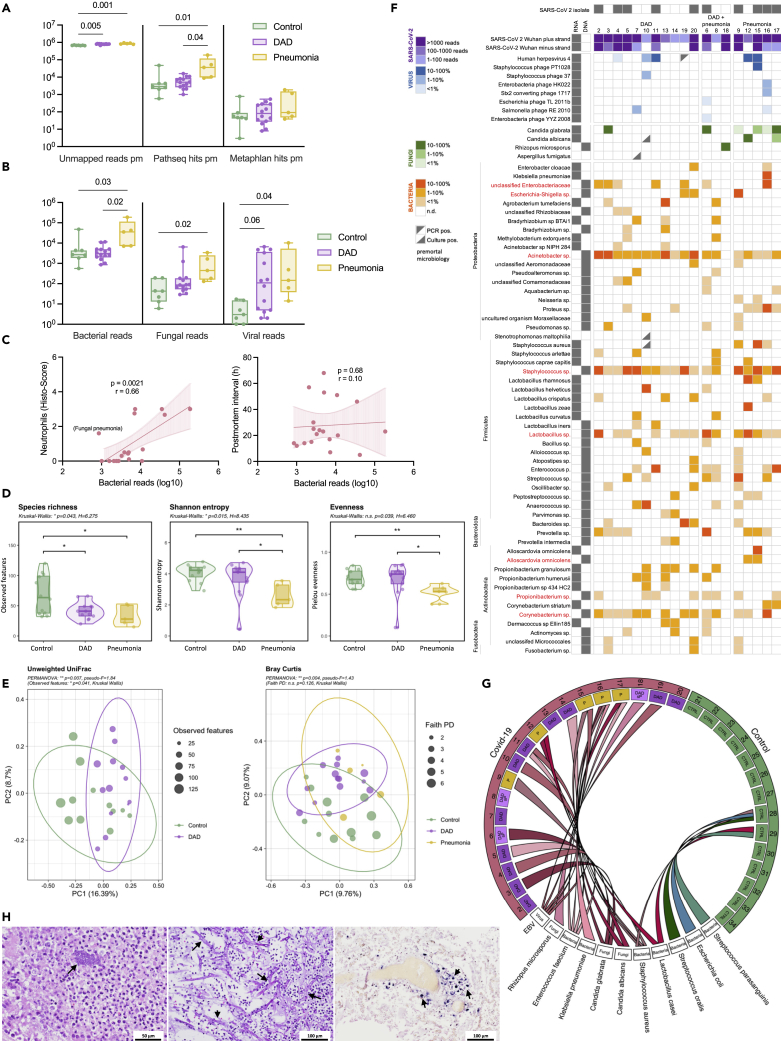
Microbiome alterations and agents of secondary infections in covid-19 lungs (A) Annotation of non-human transcripts to microbial sequences with PathSeq and MetaPhlAn, respectively (hits per million; Kruskal-Wallis test). (B) Significantly increased bacterial, fungal and viral reads in the pneumonia category of covid-19 (PathSeq annotation, Kruskal-Wallis test). (C) Bacterial reads significantly correlate with neutrophil counts but not with the post-mortem interval (Spearman correlation). (D) Richness and evenness in the bacterial component of the lung microbiome (based on the 16S rRNA gene marker; Kruskal-Wallis test). (E) Beta-diversity analysis (PCA based on unweighted UniFrac and Bray-Curtis distance) clearly separates DAD and pneumonia cases of covid-19 from controls (16S rRNA gene; PERMANOVA, Kruskal-Wallis test). (F) Summary of bacterial, fungal and viral microbes prevalent in covid-19 lungs. Shown are microbes detected by cultivation, RNA and/or DNA sequencing (red labeled taxa were also spuriously found in controls). (G) Dominant pathogens causing secondary infections in covid-19 lungs compared to controls (summary of cultivation and deep sequencing). (H) Microscopic representation (H&E) of bacterial (left, case #16) and fungal (middle, case #18) pathogens in lung tissues. Epstein-Barr virus RNA positivity in lung tissue (EBV RNA in-situ hybridization, case #11).

The lung microbiome is altered in DAD and thought to be a relevant factor for the development of secondary infections (Dickson et al., 2016; Luyt et al., 2020). RNA-seq from lung tissues was screened for microbial sequences and bacterial (16S rRNA gene) and fungal (internal transcribed spacer, ITS) marker genes were amplified to additionally specify microbial changes. On average 6573.33 ± 2552.32 (MW±SD) reads per million (rpm) per sample were not human in RNA-seq and likely of microbial origin, of those 2.02 ± 4.00% and 0.03 ± 0.05% could be clearly annotated to specific microbes with different microbial annotation pipelines (Figure 3A). Excluding SARS-CoV-2 reads, which were the dominant microbial component in several cases (range: 0.01–131218.36 rpm), bacterial sequences were dominant, significantly increased in covid-19 cases with pneumonia compared to DAD and controls. Fungal and viral sequences other than SARS-CoV-2 were also significantly enriched in covid-19 cases with pneumonia (Figure 3B). Number of bacterial reads significantly correlated with neutrophil scores suggesting that their presence is a sign of secondary infections, however, the post-mortem interval did not, precluding a strong influence of post-mortal bacterial overgrowth in our investigation (Figure 3C). Bacteria are assumed to be the dominant microbiome component in lungs (Huffnagle et al., 2017). Analysis based on the bacterial 16S rRNA gene marker showed that richness was significantly decreased in the DAD and pneumonia cases of covid-19 compared to controls indicating an overall reduced biodiversity (Figure 3D). In contrast, evenness was significantly decreased in the pneumonia group of covid-19 only, suggesting a dominance of certain taxa, possibly representing the agents of secondary infections (pairwise Kruskal-Wallis; ∗p < 0.05, ∗∗p < 0.005). Principal component analysis (PCA) clearly separated controls from covid-19 cases with DAD and cases with pneumonia indicating significantly different bacterial community compositions (Figure 3E). Lung tissues were also cultured for bacteria and fungi and both—covid-19 cases and controls—yielded cultivable microorganisms but in different quantities and taxonomic constellations (Table S7).

Finally, we integrated RNA-seq, 16S, ITS, and culture data to define dominant pathogens, most likely representing the agents of secondary infections and to account for the different samples used for microbial identifications in the light of the patchy disease representations likely impacting the microbial repertoires (Figure S10 and Table S8). Dominant pathogens were defined if they were dominant in the RNA and/or DNA data (representing >10% of microbial reads excluding SARS-CoV-2) and if they also yielded a reasonable culture growth (≥10^4^ cfu/mL). Dominant taxa were typical agents of pulmonary secondary infections like *Staphylococcus aureus*, *Enterococcus faecium*, or *Klebsiella pneumoniae*, as well as fungi like *Candida* spp. or the mold *Rhizopus microsporus* identified in one case (#18; (Zurl et al., 2021)). Often multiple pathogens were found simultaneously indicating polymicrobial infections (e.g., in case #16 wherein *K*. *pneumonia*, *S*. *aureus* and *Candida glabrata* were cultivated in reasonable amounts and were also captured by RNA-seq). In addition, 5 covid-19 cases yielded transcripts of Epstein-Barr virus (EBV), which were also detectable by RNA *in-situ* hybridization of lung tissues but not in controls (Figure 3H). EBV often emerges because of endogenous reactivation in the context of impaired immunity (Tangye et al., 2017). Control cases yielded microbial sequences and cultivable microbes in lower quantity and they often belonged to known contaminants like *Lactobacillus* sp. or *Propionibacterium* sp. (Table S8). In summary, the lung microbiome in covid-19 shows a reduced taxonomic richness but harbors a diverse spectrum of bacterial and fungal pathogens typically associated with secondary lung infections. Prominent pathogens like *S*. *aureus*, *Klebsiella* or *Candida* spp. are also known agents of secondary infection in influenza, SARS, and MERS (Klein et al., 2016; Morens et al., 2008). Notably, secondary infections were rarely detected ante-mortem in our cohort (Table S8). The presence of poly-microbial infections and the relatively high proportion of EBV positivity suggest an overall impaired immunity in covid-19 lungs.

### The lung metatranscriptome mirrors the major death categories DAD and pneumonia

**Figure 4:**
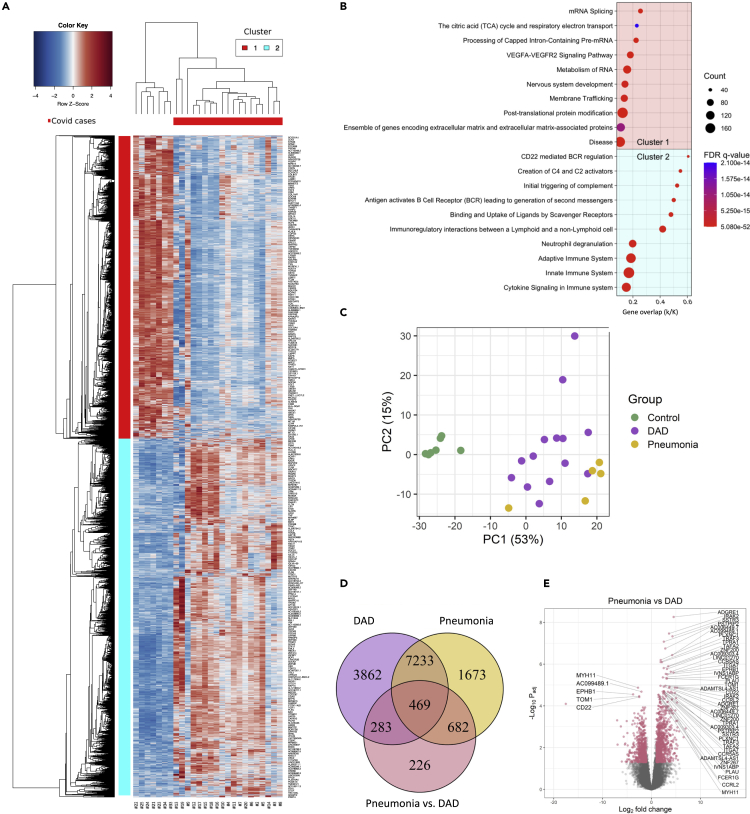
The lung metatranscriptome mirrors the major death categories DAD and pneumonia (A) Hierarchical clustering shows depleted (cluster 1) and enriched (cluster 2) genes (n = 4,547; adj. P< 0.05) in lung tissue of covid-19 cases compared to controls. (B) Gen set enrichment analysis (canonical pathways) of major depleted (top) and enriched (bottom) pathways in covid-19 lungs. (C) PCA based on differentially expressed genes clearly discriminates DAD cases and cases with secondary pneumonia of covid-19 from controls. (D) Venn diagram specifying differentially expressed genes in DAD as the major discriminator followed by pneumonia (adj. P< 0.05, LFC≥0.58). (E) Volcano plot showing the top 25 significantly deregulated genes in secondary pneumonia versus DAD. Several macrophage genes are increased.

Deep RNA-seq of lung tissue revealed 4,547 differentially expressed genes between covid-19 cases and controls (adj. p < 0.05). Hierarchical clustering indicated depleted (cluster 1) or enriched (cluster 2) genes in covid-19 compared to controls (Figure 4A). Pathway analysis indicated impaired central cellular functions within mRNA metabolism, post-translational protein modification, the respiratory chain, VEGFA signaling and extracellular matrix organization in covid-19. Enriched pathways consisted mainly of innate and adaptive immune functions, neutrophil degranulation, cytokine signaling as well as complement activation (Figure 4B). Overall, these data confirm profound and complex transcriptional alterations in covid-19 lung tissue (Delorey et al., 2021; Liao et al., 2020; Wu et al., 2020). Unsupervised principal components analysis (PCA) of differentially expressed genes clearly separated covid-19 samples on principal component 1 (PC1) from controls but also clearly separated pneumonia samples from pure DAD cases (Figure 4C). Comparison of differentially expressed genes between these major death categories indicated that the major discriminator from controls was DAD showing 3862 unique differentially expressed genes (adj. P < 0.05 and abs. LFC >= 0.58) followed by pneumonia with 1673 unique differentially expressed genes (Figure 4D). DAD and pneumonia differed by only 226 differentially expressed genes. Notably, among the top 50 differential expressed genes enriched in pneumonia cases several macrophage markers were evident, including the receptor *ADGRE1* (murine homolog F4/80) as top-hit, the interleukin-1 receptor-associated kinase-like 2 (*IRAK2*) or *PSTPIP2*, which is involved in macrophage polarization (Figure 4E). Thus, macrophages seem to be implicated in covid-19 secondary infections. In summary, deep transcriptomic analyses specified multiple dysregulated processes in covid-19, including vascular and coagulation systems, connective tissue remodeling as well as activated immunity and complement (Nie et al., 2021). Similar to histopathology, the major discriminator from controls based on gene expression was DAD followed by pneumonia likely mirroring the development of secondary infections on top of ALI caused by the virus.

### Cellular deconvolution subgroups covid-19 lung pathology

**Figure 5:**
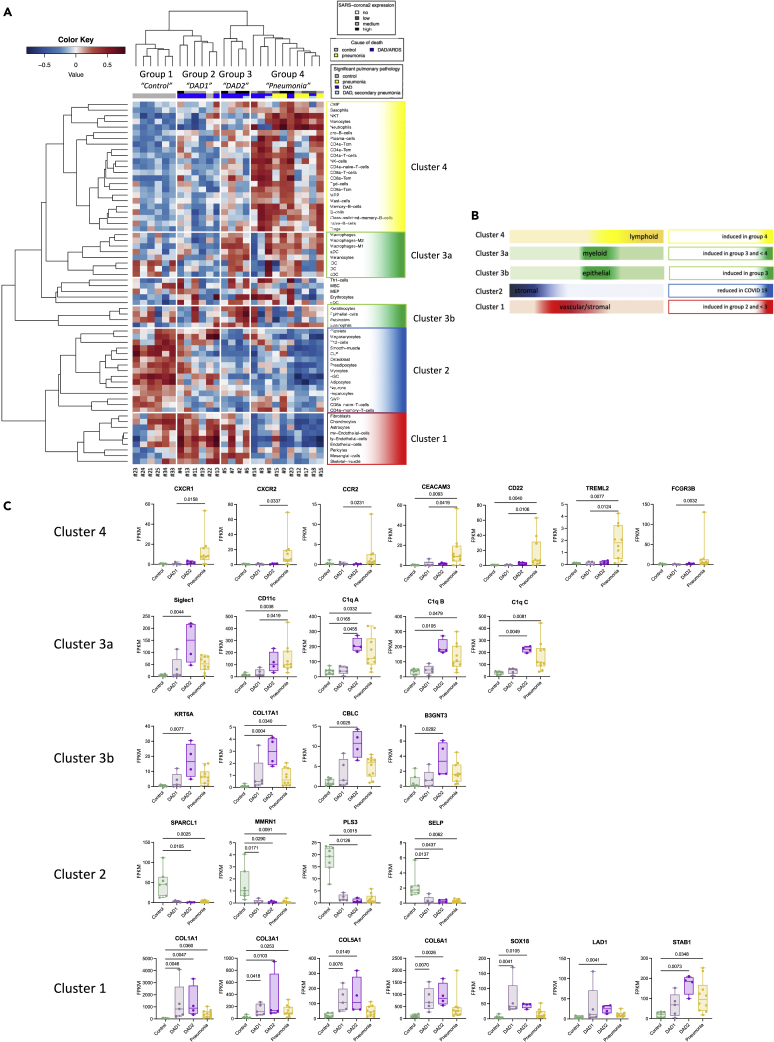
Cellular deconvolution stratifies lung pathology sub-groups (A) Hierarchical clustering based on cell-type enrichments derived from xCell analysis indicates a specific grouping of samples. (B) Scheme indicating cell clusters which discriminate different groups. (C) Top induced genes in the respective cell clusters determining the specific grouping (Kruskal-Wallis test).

Cellular compositions were inferred from RNA-seq by using xCell (Aran et al., 2017). Hierarchical clustering based on cellular compositions clearly separated samples into four distinct groups. Group 1 (“control”) consisted only of control cases and was related to group 2 (“DAD1”) consisting of covid-19 cases with pure DAD (in addition to one control case #22). Group 3 (“DAD2”) also contained DAD cases, including one sample with the histological category DAD and secondary pneumonia. This group was related to group 4 (“pneumonia”) composed of all pneumonia cases, in addition to 3 DAD cases and the two remaining DAD cases with secondary pneumonia (Figure 5A). It is noteworthy that neither disease duration nor early versus late DAD histological features or SARS-CoV-2 loads significantly correlated with a specific grouping (Figure S11). Cell types discriminating these groups showed a specific assembly (Figure 5B). Cluster 1 consisted mainly of vascular and stromal cell types like endothelial cells, pericytes and fibroblasts, enriched in “DAD1” and “DAD2”. Top enriched genes in this cluster were certain collagen genes, the vascular transcription factor *sox 18*, the basement membrane protein *ladinin-1* or the endothelial protein *stabilin-1* (Figure 5C). Cell types of cluster 2 consisted mainly of structural and stromal cells, in addition to certain immune and blood cell types and they were overall reduced in covid-19. Top down-regulated genes included the extracellular matrix proteins *sparc-like 1*, *multimerin-1* and *plastin-3* or the cell adhesion molecule *p-selectin* important for the recruitment of leukocytes typically prevalent on activated endothelial cells and platelets (Figure 5C). Together these alterations highlight the vascular and connective tissue changes emerging during DAD development (Figure 2B) (Ackermann et al., 2020; Hughes and Beasley, 2017). Cell types of cluster 3, which were dominantly induced in “DAD2” and to a lesser extent in “pneumonia” showed enrichment of myeloid (cluster 3a) and epithelial cell types (cluster 3b). Top induced genes in cluster 3a were the myeloid cell specific genes *siglec-1*, *CD11c* and complement factor *C1q*. Top induced genes in cluster 3b consisted of keratin 6A, collagen XVII, the tyrosine kinase signaling protein *cbl-c* and beta-1,3-N-acetylglucosaminyltransferase 3 (*b3gnt3*), typically expressed in epithelia and also involved in lymphocyte trafficking and homing. Cell types in cluster 4, strongly increased in “pneumonia” consisted of different leukocyte classes including B-, T-cells and (neutrophilic) granulocytes. Top induced genes consisted of the interleukin 8 receptor genes *cxcr1* and *cxcr2*, the chemokine receptor type 2 (*CCR2*), *CEACAM3*, *CD22* (B cell marker), and the cell surface receptors *TREML2* and *FCGR3B*.

In summary, cellular deconvolution clearly sub-stratified the major categories DAD and pneumonia of covid-19 lung pathology. Noteworthy, DAD subclustered into two different groups, one showing mainly induction of vascular and stromal cell elements (“DAD1”), the other dominant induction of genes related to myeloid and epithelial cells (“DAD2”), and this subgroup showed more commonalities with the pneumonia group.

### Macrophages complement c1q and immune impairment in covid-19 lungs

**Figure 6:**
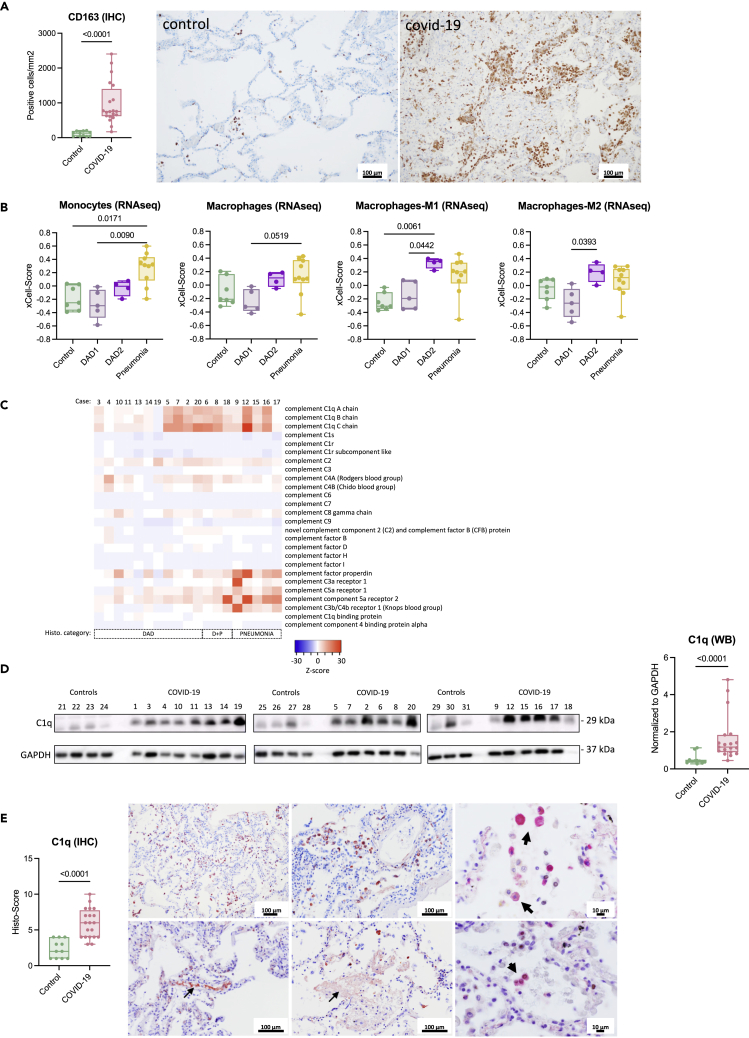
Macrophage and complement C1q induction in covid-19 lungs (A) Immunohistochemical counting of CD163 positive macrophages shows induction in covid-19 compared to controls (Mann-Whitney test). (B) Both M1 and M2 macrophages are specifically increased in “DAD2” compared to “DAD1” (grouping according to xCell analysis; Kruskal-Wallis test). (C) Heatmap of complement genes specifies C1q induction in a subgroup of DAD cases and in pneumonia. (D) C1q protein (29 kDa) is significantly increased in covid-19 lung tissue compared to controls (reference human GAPDH; Mann-Whitney test). (E) Significant induction of C1q detected by immunohistochemistry (Mann–Whitney test) and different staining patterns in covid-19 lungs; top left & middle: C1q staining of alveolar cells; top right: double immunohistochemistry staining (red: C1q, nuclear black: TTF-1) shows C1q staining of alveolar macrophages; bottom left: intravascular C1q staining; bottom middle: free C1q specific staining of proteinaceous fluid in the alveolar space; bottom right: double immunohistochemistry staining (red: C1q, nuclear black: TTF-1) shows C1q staining of pneumocytes (TTF-1 positive).

Myeloid cells including macrophages play a central role in the pathogenesis of DAD (Chen et al., 2020; Fan and Fan, 2018; Huang et al., 2018), and bronchalveolar lavage fluids (BALFs) of patients with severe covid-19 reveal high proportions of macrophages (Liao et al., 2020; Wang et al., 2020). We confirmed significantly increased macrophages in covid-19 lungs by CD163 immunohistochemistry, depicting a M2-type macrophage marker (Figure 6A), corroborating a recent proteomic study wherein CD163 was found among the most induced proteins in lungs and spleens derived from covid-19 autopsies (Nie et al., 2021). Deconvolution indicated both M1- and M2-type macrophages significantly enriched predominantly in “DAD2” whereas monocytes were mainly induced in the “pneumonia” group (Figure 6B). Increased CD163 positive macrophages gathering around virus positive cells were recently shown also in a macaque model of SARS-CoV infection, indicating that infected pneumocytes may lead to macrophage recruitment in coronavirus infections (Liu et al., 2019).

Among the most discriminative genes between DAD subtypes we found complement factor *C1q* dominantly induced in “DAD2” (Figure 5C). Complement activation is implicated in DAD pathogenesis and linked to severe covid-19 (Holter et al., 2020; Java et al., 2020; Perico et al., 2021). Other complement factors showed no discriminative expression pattern between pathological subgroups in our cohort, except certain complement receptors and properdin mainly induced in pneumonia cases (Figure 6C). C1q levels did not correlate with survival times of patients (Figure S12). Western blots generated from extracts of lungs confirmed significantly increased C1q protein (Figure 6D). A major source of C1q are macrophages corroborated also by a recent single-cell transcriptomic analysis of covid-19 lungs (Figure S13) (Lu et al., 2008; Xu et al., 2020) suggesting a strong connection between macrophages and complement C1q in covid-19 (Carvelli et al., 2020). Immunohistochemical analysis of lung tissues with a C1q specific antibody showed staining of the vasculature, the interstitial and alveolar space but also of alveolar cells including macrophages and pneumocytes, indicating a multifaceted deposition of C1q in the context of covid-19 in our series (Figure 6E).

**Figure 7:**
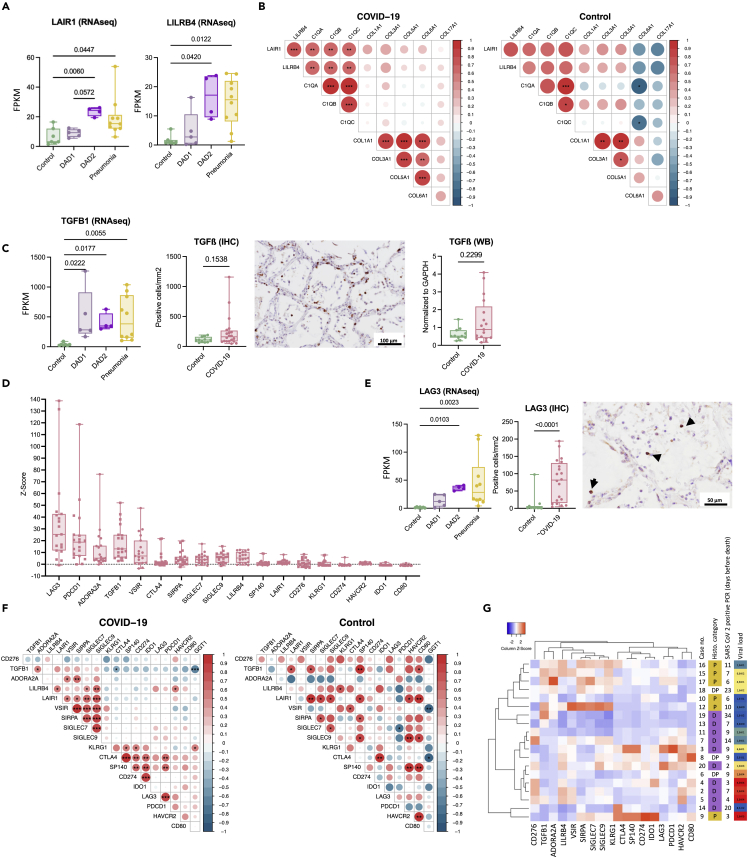
Signatures of immune-impairment in covid-19 lungs (A) The tolerogenic leukocyte receptors *LAIR-1* and *LILRB4* are mainly induced in “DAD2” and “pneumonia” (Kruskal–Wallis test). (B) Spearman correlation of RNA expression of *LAIR-1* and *LILRB4* with *C1q* chains and induced collagen types (Spearman r; p∗<0.05 to p∗∗∗<0.001). (C) Significant induction of *TGFβ1* transcription (Kruskal-Wallis test). Protein measurement by immunohistochemistry and western blotting does not reveal a significant difference of covid-19 lungs to controls (Mann-Whitney test). (D) Strong induction of immune checkpoint inhibitors in covid-19 (order according to z-score). (E) *LAG3* transcriptional induction (Kruskal-Wallis test) and increased lymphocyte staining with LAG3 immunohistochemistry in covid-19 lung tissue (Mann-Whitney test). (F) Simultaneous transcriptional induction of immune checkpoint inhibitors in covid-19 lungs compared to controls (Pearson correlation; p∗<0.05, p∗∗∗<0.001). (G) Hierarchical clustering of immune checkpoint inhibitors in covid-19 cases shows a different grouping of samples with high viral loads (transcript abundance) versus samples with the histological pneumonia category (clustering: average linkage; distance measure: Pearson).

C1q is the initiating component of the classical complement cascade but exhibits also immune regulatory functions. It induces the development of pro-resolving M2 type macrophages and is involved in the clearance of apoptotic and necrotic cells, which are highly increased in covid-19 lungs (Bohlson et al., 2014; Li et al., 2020b; Mulay et al., 2021). In this process C1q binds to cellular break-down products and is subsequently recognized by phagocyte receptors like the leukocyte-associated immunoglobulin-like receptor 1 (LAIR-1; syn.: CD305) conferring uptake and triggering a tolerogenic state in the phagocyte (Son et al., 2015; Thielens et al., 2017). As shown by a recent single-cell transcriptomics analysis of covid-19 lungs, *LAIR-1* is mainly present in macrophages (Figure S14) (Delorey et al., 2021). LAIR-1 together with LILRB4 (leukocyte immunoglobulin-like receptor subfamily B member 4; syn.: ILT3) belong to immunoglobulin-like receptors recognizing collagen domains such as present in C1q, thereby inhibiting immune activation (Lebbink et al., 2006; Son et al., 2012). RNA-seq confirmed significant induction of *LAIR-1* and *LILRB4* dominantly in “DAD2” followed by “pneumonia” (Figure 7A). Expressions of all 3 *C1q* polypeptide chains (*A*, *B, & C*) significantly correlated with *LAIR-1* and *LILRB4* expression but not with induced collagens (Figures 7B and S15). This might suggest a functional link between C1q and the immune inhibitory receptors LAIR-1 and LILRB4.

Because the development of secondary infections is likely driven by local immune-impairment we screened for other anti-inflammatory markers. TGF-β1 is a key factor in the development and healing response of ALI and also implicated in covid-19 lung pathology (Peters et al., 2014; Vaz dePaula et al., 2021). *TGF-β1* transcription was significantly increased in covid-19, showing a huge variability, however, TGF-β1 protein measured by immunohistochemistry and western blotting showed no significant induction compared to controls (Figures 7C and S16). Because several immune and non-immune cell types are able to produce TGF-β1, the observed variability in expression might reflect the temporal heterogeneity of lung pathologies in our cohort. Induction of certain inhibitory immune-checkpoints is reported in covid-19 (Bobcakova et al., 2021; Diao et al., 2020; Files et al., 2021; Jeannet et al., 2020; Li et al., 2020a). We confirmed transcriptional induction of several inhibitory immune-checkpoints in covid-19 lungs (*LAG3*, *PDCD1*, *ADORA2A*, *VSIR*, *CTLA4*, *SIRPA*, *LAIR1*, *SIGLEC9*, *LILRB4*, *SIGLEC7*, and *HAVCR2*), whereas some showed no induction (*CD276*, *SP140*, *IDO1*, *KLRG1*, and *CD274*) or were even reduced (*GGT1* and *CD80*) compared with controls (Figures 7D and S17). Of note, the top induced inhibitory immune-checkpoint was found to be *LAG3* (lymphocyte-activation gene 3; syn.: CD223), dominantly induced in “DAD2” and “pneumonia” based on RNA-seq, which was also confirmed by immunohistochemistry wherein mainly lymphocytes showed strong staining signals (Figures 7E and S18). LAG3 was recently described as a major increased factor in a plasma proteomic study of severe covid-19 (Filbin et al., 2021). During immune exhaustion multiple inhibitory receptors act often in synergy amplifying immune impairment, like LAG3 and PD-1 co-induced during chronic viral infections (Blackburn et al., 2009). We confirmed synergistic induction of several inhibitory immune-checkpoints in covid-19 lungs, which showed a different costimulatory pattern compared to controls (Figure 7F). Of interest, co-expression patterns discriminated cases with high viral loads (*KLRG1*, *CTLA4*, *SP140*, *CD274*, *IDO1*, *LAG3*, *PDCD1*, *HAVCR2*, and *CD80*) from samples with pneumonia (*CD276*, *TGFB1*, *ADORA2A*, *LILRB4*, *LAIR1*, *VSIR*, *SIRPA*, *SIGLEC7*, and *SIGLEC9*) suggesting a divergent pattern of induction of inhibitory immune-checkpoints during the course of covid-19 lung pathology (Figure 7G). In summary, these data highlight that multiple pillars of immune impairment act in severe covid-19, leading to a reduced antimicrobial defense in lungs driving the development of secondary infections. The molecular dissection of cell types and immune inhibitory signals might enable the development of specific measures counteracting this potentially lethal complication.

## Discussion

We performed a systematic autopsy study of 20 consecutive covid-19 cases and 14 controls to gain unbiased information about lethal disease courses from the early pandemic. Integration of autopsy, cultivation and deep sequencing provided important clues about host and microbial factors involved in the development of secondary infections as a major sequel of lethal covid-19. Thus, our study might serve as a blue-print for a “holistic” autopsy approach tempting to gain relevant pathophysiological insights from a newly emerging disease (Layne et al., 2022). Viral genotyping facilitated directly from autopsy material provided epidemiologic clues about transmission and captured already early events of viral genetic adaptation. Of note, a significant proportion of corpses yielded cultivable SARS-CoV-2 indicating that autopsy might facilitate virus spread and that special safety requirements should be applied during post-mortem examinations of covid-19 patients (Loibner et al., 2021). Our investigation showed that covid-19 lung pathology is multifaceted and that a major discriminator of lethal courses is DAD and the presence of secondary infections. This was evident by histology but also mirrored by the deep transcriptomic analysis and microbiology. Secondary infections are reported to develop in up to 42% of patients with covid-19 (Buehler et al., 2021). Notably, DAD caused by the virus itself and secondary infections are chronologically divergent and provoke overtly different host reactions. It is also noteworthy that SARS-CoV-2 infection alone might not trigger prominent neutrophil recruitment to the lung at all and neutrophil signatures found in recent covid-19 studies might likely already indicate secondary infections (Liao et al., 2020; Melms et al., 2021; Nie et al., 2021; Wauters et al., 2021; Xu et al., 2020). Thus, it is important to seek for a proper pre-classification of tissue samples based on histology to omit wrong conclusions in molecular down-stream analyses.

The resident lung microbiome is a relevant factor in the pathogenesis of lung infections and reported to be altered in sepsis and DAD (Dickson et al., 2016; Luyt et al., 2020). Secondary lung infections are also complicating influenza, SARS and MERS, wherein bacteria like *S*. *aureus* or *Klebsiella* spp. and fungi such as *Candida* or *Aspergillus* spp. are found (Klein et al., 2016; Morens et al., 2008). Such microbial agents were also present in our cohort. Curiously, the mechanistic understanding why secondary infections develop on top of viral infections is still limited. We could not identify any associated clinical parameter clearly correlated with secondary infections but showed that lung immunity is impaired in covid-19, which might drive these infections. This finding was also underscored by the presence of polymicrobial infections and EBV indicative for a general decreased immunity (Tangye et al., 2017). Typical comorbidities of covid-19, like chronic kidney disease or diabetes, are already signified by a lowered immunity increasing the infection risk (Carey et al., 2018; Syed-Ahmed and Narayanan, 2019). Moreover, ICU admission and mechanical ventilation are established risk factors for the development of pneumonias (Wu et al., 2019). Thus, secondary lung infections in covid-19 could originate from different sources including direct immune challenge by SARS-CoV-2, the underlying condition and medical interventions (Callender et al., 2020). Curiously, the majority of our patients received antibiotics, which failed to effectively protect against secondary infections. This circumstance might have been influenced by underdeveloped antibiotic regimens or ineffective substances (e.g., chloroquine; (Axfors et al., 2021)) given during the early pandemic but also from concomitant therapeutic immunosuppression (e.g., corticosteroids) altogether modulating the infection risk on an individual base. Notably, ICU admission and intubation, however, were generally associated with lower neutrophil scores. Because all intubated patients received antibiotics in our series, this therapy might have delayed secondary pneumonia development.

Severe cases of covid-19 typically present with high inflammatory markers complicating the distinction between severe courses with pure DAD or bacterial or fungal secondary infections. As in our cohort, hospitalized covid-19 patients are empirically treated with broad-spectrum antibiotics but efficacy of this therapy is still debated (Sieswerda et al., 2021). Because patients frequently need prolonged hospitalizations and respiratory support, unnecessary antibiotic therapy also likely increases the risk of hospital-acquired pneumonias caused by resistant bacteria. On a population level increased antibiotic usage likely leads to rising antimicrobial resistance further complicating management of the pandemic (Chong et al., 2021; Sopirala, 2021). Noteworthy, antibiotic challenge of the resident lung microbiome might also impact secondary infection development. In analogy to the GI tract wherein depletion of the resident microbiome potentially provokes overgrowth of pathogens (e.g., in *C*. *difficile* colitis; (Chang et al., 2008)), similar mechanisms might happen in the respiratory tract. Notably, we detected a significant reduced biodiversity in lungs of our covid-19 patients which was likely influenced by antibiotics. Single reports indicate a rise of secondary infections in the second pandemic wave compared to the initial phase indicating a fluctuating picture of secondary infections (Fortarezza et al., 2022; Hedberg et al., 2022). This development might have been influenced by several factors including a changed epidemiology of patients (different comorbidities and ages), seasonal effects, which seem to be particularly important for the development of fungal pneumonias but also changes in clinical practice (Ayzac et al., 2016; Group et al., 2021).

Immune exhaustion seems to follow the systemic immune hyperactivation in severe covid-19 and myeloid cells, which are important for the recognition of virus infected cells, are key to initiate the proinflammatory response (McGonagle et al., 2020; Merad and Martin, 2020). Recent single-cell transcriptomic studies of covid-19 patients identified myeloid cells as a major induced cell type in BALF specimens with high proportions of proinflammatory macrophages (Liao et al., 2020; Melms et al., 2021). Generally, M1-type macrophages dominate early DAD, whereas later DAD stages show increased M2-types involved in tissue repair with immunosuppressive features (Huang et al., 2018). Thus, later (organizing) phases of DAD might be specifically prone to acquire secondary infections. Respiratory failure in covid-19 is linked to strong complement activation (Holter et al., 2020; Java et al., 2020; Messner et al., 2020), which likely occurs when the disease progresses (Nienhold et al., 2020). Extensive deposition of complement factors, including C1q, in vessels and epithelial cells of lungs and skin was reported in covid-19 (Macor et al., 2021; Magro et al., 2020). Notably, the SARS-CoV-2 spike-protein might directly activate complement via the alternative pathway (Yu et al., 2020). Complement in covid-19 is currently discussed mainly in the context of endothelial injury and fibrin-clot formation (Perico et al., 2021). Our study suggests another pathophysiological role, wherein C1q and macrophages might perpetuate immune impairment. Immune complexes formed by viral antigens and antibodies can activate factor C1 as shown in SARS-CoV infection (Yang et al., 2005). C1q is involved in the clearance of apoptotic and necrotic cells by phagocytes, a process termed efferocytosis (Doran et al., 2020). Apoptosis and necrosis are prominent in covid-19 lungs (Filbin et al., 2021; Li et al., 2020b; Mulay et al., 2021). During efferocytosis suppression of overwhelming inflammation is important and phagocytes involved in this process are producing anti-inflammatory cytokines. Therefore, C1q binds to molecules released from apoptotic and necrotic cells (e.g., phosphatidylserine, nucleic acids, etc.) and these complexes are recognized by receptors present on phagocytes, like LAIR-1, conferring uptake and inducing a tolerogenic state (Bohlson et al., 2014; Lu et al., 2008; Son et al., 2015; Thielens et al., 2017). Noteworthy, binding of C1q to LAIR-1 on plasmacytoid DCs restricts the production of type I interferons impairing antiviral defense, which also occurs in covid-19 (Son et al., 2012, 2015). The “DAD2” subtype in our study shows increased macrophages, C1q and LAIR-1 and might therefore represent cases with a lowered immune tone prone for the development of secondary infections. Overall the progression of early (exudative) DAD into late (fibrotic) DAD indicates healing of ALI characterized by significant connective tissue remodeling and a reduced inflammatory tone (Margaroli et al., 2021; Matthay et al., 2019). Immune suppressive factors such as TGF-β1 are known to be involved in this process and also LAIR-1 and LILRB4 recognizing collagens or collagen-like proteins might act anti-inflammatory during this disease phase (Fernandez and Eickelberg, 2012; Fouet et al., 2021; Paavola et al., 2021; Son et al., 2012; Tomic et al., 2021). Moreover, the synergistic induction of several tolerogenic factors including inhibitory immune-checkpoints (Bobcakova et al., 2021; Diao et al., 2020; Filbin et al., 2021; Files et al., 2021; Hadjadj et al., 2020; Jeannet et al., 2020; Li et al., 2020a) and increased (apoptotic) cell death of immune-cells (Cizmecioglu et al., 2021; Feng et al., 2020) altogether perpetuate immune failure in covid-19.

### Limitations of the study

The limitations of our descriptive study are that causalities cannot be directly inferred and that the relatively small cohort cannot reveal the entire picture of severe covid-19 and associated secondary infections. Varying clinical courses and different comorbidities might also have influenced our findings. In addition, treatment of covid-19 has changed since the early pandemic, thus, current severe courses and developing sequels might also have changed. We also cannot be sure whether the two described forms of DAD might represent just a spectrum of pathophysiological states or are specific pathotypes. Moreover, post-mortem effects like RNA degradation might have introduced additional noise in our investigation. Nevertheless, we found autopsy complemented with microbiology and molecular measures as a powerful tool to gain relevant clues about covid-19 pathophysiology. Of importance, there exists an obvious knowledge gap in the understanding of the molecular mechanisms driving the development of secondary infections on top of in viral lung diseases. This should initiate further studies to understand the molecular pathways in more detail and to unravel chronological phases of immuno-suppression which could also lead to development of rational therapies counteracting this sequel not only in covid-19. For these investigations, autopsy specimens and associated molecular data might serve as a valuable resource.

## STAR★Methods

### Key resources table

**Table:** 

REAGENT or RESOURCE	SOURCE	IDENTIFIER
**Antibodies**		

Anti-SARS-CoV-2 nucleoprotein monoclonal rabbit antibody	Sino Biological	Cat# 40143-R019; clone: 019; RRID:AB_2827973
Anti-CD68 monoclonal mouse antibody	Ventana Medical Systems	Cat# 790-2931; clone: KP-1; RRID:AB_2335972
Anti-CD163 monoclonal mouse antibody	Ventana Medical Systems	Cat# 760-4437; clone: MRQ-26; RRID:AB_2335969
Anti-TTF1 monoclonal mouse antibody	Cell Marque	Cat# 343M-96; clone: 8G7G3/1; RRID:AB_1158937
Anti-TGFß1 polyclonal rabbit antibody	Santa Cruz Biotechnology	Cat# SC-146; RRID:AB_632486
Anti-TGFß polyclonal rabbit antibody	Cell Signaling Technology	Cat# 3711; RRID:AB_2063354
Anti-LAG3 polyclonal rabbit antibody	Abcam	Cat# ab180187; clone: EPR4392(2); RRID:AB_2888645
Anti-C1q polyclonal rabbit antibody	Agilent	Cat# A0136; RRID:AB_2335698
Anti-GAPDH monoclonal rabbit antibody	Cell Signaling Technology	Cat# 2118; clone: 14C10; RRID:AB_561053
HRP-linked ECL Anti-Rabbit IgG	GE Healthcare	Cat# NA934; RRID:AB_772206
Dako REAL TM EnVision TM HRP rabbit/mouse detection-system	Agilent	Cat# K5007; RRID:AB_2888627
ultraView DAB detection-system	Roche	Cat# 760-500; RRID:AB_2753116

**Bacterial and virus strains**		

SARS-CoV-2 strains from study	This study	N/A
Bacterial and fungal strains from study	This study	N/A

**Biological samples**		

Autopsy tissue and body fluid samples	This study	N/A
Respiratory tract swabs	This study	N/A

**Chemicals, peptides, and recombinant proteins**		

Thioglycollate broth	Oxoid	Cat# CM0173
OptiPro SFM medium	Gibco	Cat# 12309019
L-Glutamine	Gibco	Cat# 11539876
Penicillin-Streptomycin (10.000 U/ml)	Gibco	Cat# 11548876
TRIzol®	Invitrogen	Cat# 15596026
RIPA buffer	Sigma	Cat# R0278
Pefabloc	Roche	Cat# 11429868001
cOmplete^TM^ Mini	Merck	Cat# 11836153001
PhosSTOP^TM^	Roche	Cat# 4906845001
Laemmli buffer	Bio-Rad	Cat# 1610737EDU

**Critical commercial assays**		

MagNA Lyser green beads tubes	Roche	Cat# 03358941001
Maxwell 16 LEV simplyRNA blood kit	Promega	Cat# AS1310
QIAamp Viral RNA Mini Kit	Qiagen	Cat# 221413
High-Capacity cDNA Reverse Transcription Kit with RNase inhibitor	Applied Biosystems	Cat# 4374966
SuperScript III One-Step RT-PCR System with Platinum Taq High Fidelity DNA Polymerase mastermix	ThermoFisher	Cat# 12574018
SYBR Green PCR Mastermix	Applied Biosystems	Cat# 4309155
Ampure XP beads	Beckman Coulter	Cat# A63881
NEBNext Fast DNA Fragmentation & Library Prep Set for Ion Torren kit	New England Biolabs	Cat# E6285L
KAPA RNA HyperPrep Kit with RiboErase (HMR) for Illumina® platforms	KAPABIOSYSTEMS	Cat# KR1351
16s Complete PCR Mastermix kit	Molzym	Cat# S-020-0250
QiaQick gel extraction kit	Qiagen	Cat# 28706X4
Ponceau S solution	Sigma	Cat# P7170
ECL Select Western Blot Reagent	Amersham	Cat# 12644055
RNAlater	ThermoFisher	Cat# AM7024

**Deposited data**		

16S rRNA gene-, ITS- and RNAseq data	European nucleotide archive (ENA)	Acc. no. PRJEB45873

**Experimental models: Cell lines**		

Vero CCL-81 cells	European Collection of Authenticated Cell Cultures	ECACC 84113001

**Oligonucleotides**		

RdRp_SARSr-F GTGARATGGTCATGTGTGGCGG	(Corman et al., 2020)	N/A
RdRp_SARSr-P2 FAM-CAGGTGGAACCTCATCAGGAGATGC-BBQ	(Corman et al., 2020)	N/A
RdRp_SARSr-R CARATGTTAAASACACTATTAGCATA	(Corman et al., 2020)	N/A
N_Sarbeco_F CACATTGGCACCCGCAATC	(Corman et al., 2020)	N/A
N_Sarbeco_P FAM-ACTTCCTCAAGGAACAACATTGCCA-BBQ	(Corman et al., 2020)	N/A
N_Sarbeco_R GAGGAACGAGAAGAGGCTTG	(Corman et al., 2020)	N/A
2019-nCoV_N1-F GACCCCAAAATCAGCGAAAT	https://www.cdc.gov/coronavirus/2019-ncov/lab/rt-pcr-panel-primer-probes.html	N/A
2019-nCoV_N1-R TCTGGTTACTGCCAGTTGAATCTG	https://www.cdc.gov/coronavirus/2019-ncov/lab/rt-pcr-panel-primer-probes.html	N/A
2019-nCoV_N2-F TTACAAACATTGGCCGCAAA	https://www.cdc.gov/coronavirus/2019-ncov/lab/rt-pcr-panel-primer-probes.html	N/A
2019-nCoV_N2-R GCGCGACATTCCGAAGAA	https://www.cdc.gov/coronavirus/2019-ncov/lab/rt-pcr-panel-primer-probes.html	N/A
2019-nCoV_N3-F GGGAGCCTTGAATACACCAAAA	https://www.cdc.gov/coronavirus/2019-ncov/lab/rt-pcr-panel-primer-probes.html	N/A
2019-nCoV_N3-R TGTAGCACGATTGCAGCATTG	https://www.cdc.gov/coronavirus/2019-ncov/lab/rt-pcr-panel-primer-probes.html	N/A
RP-F AGATTTGGACCTGCGAGCG	https://www.cdc.gov/coronavirus/2019-ncov/lab/rt-pcr-panel-primer-probes.html	N/A
RP-R GAGCGGCTGTCTCCACAAGT	https://www.cdc.gov/coronavirus/2019-ncov/lab/rt-pcr-panel-primer-probes.html	N/A
GAPDH_f CCTCCACCTTTGACGCT	https://www.cdc.gov/coronavirus/2019-ncov/lab/rt-pcr-panel-primer-probes.html	N/A
GAPDH_r TTGCTGTAGCCAAATTCGTT	https://www.cdc.gov/coronavirus/2019-ncov/lab/rt-pcr-panel-primer-probes.html	N/A
CoV_gen_f1 TAAAGGTTTATACCTTCCCAGG	This study	N/A
CoV_gen_r1 CAGATGTGAACATCATAGCATC	This study	N/A
CoV_gen_f2 AAAGAGCTATGAATTGCAGACACC	This study	N/A
CoV_gen_r2 GGAGGGTAGAAAGAACAATACA	This study	N/A
CoV_gen_f3 GATGCTATGATGTTCACATCTG	This study	N/A
CoV_gen_r3 CAGAATCTGGATGAAGATTGCCAT	This study	N/A
CoV_gen_f4 TGTATTGTTCTTTCTACCCTCC	This study	N/A
CoV_gen_r4 CTCCATCCAAATAAGTTGGACCAA	This study	N/A
CoV_gen_f5 ATGGCAATCTTCATCCAGATTCTG	This study	N/A
CoV_gen_r5 CACATCACCATTTAAGTCAGGGAA	This study	N/A
CoV_gen_f6 TTGGTCCAACTTATTTGGATGGAG	This study	N/A
CoV_gen_r6 CACTCTGCAACTAAGCCAAA	This study	N/A
CoV_gen_f7 TTCCCTGACTTAAATGGTGATGTG	This study	N/A
CoV_gen_r7 GCCAGTAACTTCTATGTCAGATTG	This study	N/A
CoV_gen_f8 TTTGGCTTAGTTGCAGAGTG	This study	N/A
CoV_gen_r8 CACTAGTAGATACACAAACACCAG	This study	N/A
CoV_gen_f9 CAATCTGACATAGAAGTTACTGGC	This study	N/A
CoV_gen_r9 CCAGCCTGTACCAAGAAATTA	This study	N/A
CoV_gen_f10 CTGGTGTTTGTGTATCTACTAGTG	This study	N/A
CoV_gen_r10 CCAACCATGTCATAATACGCAT	This study	N/A
CoV_gen_f11 TAATTTCTTGGTACAGGCTGG	This study	N/A
CoV_gen_r11 CCAACTTACGTTGCATGGCTG	This study	N/A
CoV_gen_f12 ATGCGTATTATGACATGGTTGG	This study	N/A
CoV_gen_r12 GGATGATCTATGTGGCAACGG	This study	N/A
CoV_gen_f13 CAGCCATGCAACGTAAGTTGG	This study	N/A
CoV_gen_r13 GGTGGTATGTCTGATCCCAATATT	This study	N/A
CoV_gen_f14 CCGTTGCCACATAGATCATCC	This study	N/A
CoV_gen_r14 GCATGTTAGGCATGGCTCTATCA	This study	N/A
CoV_gen_f15 AATATTGGGATCAGACATACCACC	This study	N/A
CoV_gen_r15 GGTCGTAACAGCATTTACAA	This study	N/A
CoV_gen_f16 TGATAGAGCCATGCCTAACATGC	This study	N/A
CoV_gen_r16 GTCTCAGGCAATGCATTTAC	This study	N/A
CoV_gen_f17 TTGTAAATGCTGTTACGACC	This study	N/A
CoV_gen_r17 GCTTCTCTAGTAGCATGACACCC	This study	N/A
CoV_gen_f18 GTAAATGCATTGCCTGAGAC	This study	N/A
CoV_gen_r18 CACATGGACTGTCAGAGTAATAGA	This study	N/A
CoV_gen_f19 GGGTGTCATGCTACTAGAGAAGC	This study	N/A
CoV_gen_r19 CACTTAGATGAACCTGTTTGCGC	This study	N/A
CoV_gen_f20 TCTATTACTCTGACAGTCCATGTG	This study	N/A
CoV_gen_r20 GACTAGAGACTAGTGGCAATAA	This study	N/A
CoV_gen_f21 GCGCAAACAGGTTCATCTAAGTG	This study	N/A
CoV_gen_r21 GCAAATCTGGTGGCGTTAAA	This study	N/A
CoV_gen_f22 TTATTGCCACTAGTCTCTAGTC	This study	N/A
CoV_gen_r22 GAGGAGAATTAGTCTGAGTCT	This study	N/A
CoV_gen_f23 TTTAACGCCACCAGATTTGC	This study	N/A
CoV_gen_r23 GCTCTGATTTCTGCAGCTCTAATT	This study	N/A
CoV_gen_f24 AGACTCAGACTAATTCTCCTC	This study	N/A
CoV_gen_r24 CCTTGGAGAGTGCTAGTTGCC	This study	N/A
CoV_gen_f25 AATTAGAGCTGCAGAAATCAGAGC	This study	N/A
CoV_gen_r25 GGCATAGGCAAATTGTAGAAGACA	This study	N/A
CoV_gen_f26 GGCAACTAGCACTCTCCAAGG	This study	N/A
CoV_gen_r26 GTGAAACTGATCTGGCACGTAACT	This study	N/A
CoV_gen_f27 TGTCTTCTACAATTTGCCTATGCC	This study	N/A
CoV_gen_r27 CCATAGGGAAGTCCAGCTTCTG	This study	N/A
CoV_gen_f28 AGTTACGTGCCAGATCAGTTTCAC	This study	N/A
CoV_gen_r28 GTCCTCCCTAATGTTACACA	This study	N/A
CoV_gen_f29 CAGAAGCTGGACTTCCCTATGG	This study	N/A
CoV_gen_r29 TTTGTATGCGTCAATATGCTT	This study	N/A
CoV_gen_f30 TGTGTAACATTAGGGAGGAC	This study	N/A
CoV_gen_r30 TTTGTCATTCTCCTAAGAAGC	This study	N/A
16S_515_f TGCCAGCAGCCGCGGTAA	(Maiwald 2011)	
16S_806_r GGACTACCAGGGTATCTAAT	(Maiwald 2011)	
ITS1 TCCGTAGGTGAACCTGCGG	(Halwachs et al., 2017)	
ITS2 GCTGCGTTCTTCATCGATGC	(Halwachs et al., 2017)	

**Software and algorithms**		

R (v4.1)	https://www.R-project.org/	N/A
GISAID SARS-CoV-2 (hCoV-19) database	GISAID	https://www.gisaid.org
clustalw (v2.1)	(Larkin et al., 2007)	ftp://ftp.ebi.ac.uk/pub/software/clustalw2/
figtree (v1.4.4)		http://tree.bio.ed.ac.uk/software/figtree/
STAR	(Dobin et al., 2013)	https://github.com/alexdobin/STAR
bowtie2-2.4.1	(Langmead and Salzberg 2012)	http://bowtie-bio.sourceforge.net/bowtie2/index.shtml
HTSeq (v0.12.4)	G Putri, S Anders, PT Pyl, JE Pimanda, F Zanini Analysing high-throughput sequencing data in Python with HTSeq 2.0 https://doi.org/10.1093/bioinformatics/btac166(2022)	https://htseq.readthedocs.io/en/master/
xCell	(Aran et al., 2017)	https://github.com/dviraran/xCell
edgeR	(Robinson et al., 2010)	https://bioconductor.org/packages/release/bioc/html/edgeR.html
Gene set enrichment analysis online tool	(Subramanian et al., 2005)	https://www.gsea-msigdb.org/gsea/msigdb/annotate.jsp
Single-cell atlas database SCovid	(Qi et al., 2022)	http://bio-annotation.cn/scovid/
MetaPhlAn2 (v2.6.0)	(Segata et al., 2012)	https://github.com/biobakery/MetaPhlAn2
Pathseq (GATK v4.1.0.0)	(Kostic et al., 2011)	https://github.com/broadinstitute/gatk
QIIME2 (v. 2020.6)	(Bolyen et al., 2019)	https://qiime2.org/
LEfSe	(Segata et al., 2011)	https://www.bioconductor.org/packages/release/bioc/html/lefser.html
DADA2	(Callahan et al., 2016)	https://bioconductor.org/packages/release/bioc/html/dada2.html
UNITE reference database	(Nilsson et al., 2018) Nilsson RH, Larsson K-H, Taylor AFS, Bengtsson-Palme J, Jeppesen TS, Schigel D, Kennedy P, Picard K, Glöckner FO, Tedersoo L, Saar I, Kõljalg U, Abarenkov K. 2018. The UNITE database for molecular identification of fungi: handling dark taxa and parallel taxonomic classifications. Nucleic Acids Research, https://doi.org/10.1093/nar/gky1022	https://unite.ut.ee/
SILVA reference database	(Quast et al., 2013)	https://www.arb-silva.de/
bcftools (v1.3.1)		http://github.com/samtools/bcftools
Incscape (v0.92)		https://inkscape.org/de/release/inkscape-0.92/
fastx (v0.0.13)		http://hannonlab.cshl.edu/fastx_toolkit/
seqclean		https://sourceforge.net/projects/seqclean/
samtools	(Danecek et al., 2021)	http://www.htslib.org/
GraphPad Prism^TM^		https://www.graphpad.com/scientific-software/prism/
ImageJ		https://imagej.nih.gov/ij/index.html
BioRender		https://biorender.com/

**Other**		

eSwab	Copan	Cat# 80490CEA
Inform EBER Epstein Barr Virus early RNA kit	Ventana	Cat# 800-2824
ISH invers blue detection-system	Ventana	Cat# 800-092
Genbox anaer	bioMérieux	Cat# 45534
Blood agar	BD Diagnostics	Cat# 256506
MacConkey agar	BD Diagnostics	Cat# 215197
Chocolate agar	BD Diagnostics	Cat# 257456

### Resource availability

#### Lead contact

Further information and requests for resources and reagents should be directed to and will be fulfilled by the lead contact, Gregor Gorkiewicz (gregor.gorkiewicz@medunigraz.at)

#### Materials availability

There are restrictions to the availability of tissue samples, viral, bacterial and fungal strains generated by the study (e.g. ethics, biosafety). Inquiries should be directed to the lead contact.

### Experimental model and subject details

Twenty consecutive covid-19 patients which deceased at the first pandemic peak in our institution (23/3/2020 and 26/4/2020) were post-mortally examined. In addition, 14 age-matched non-covid-19 controls which deceased within the same time period were included for subsequent analyses. They were selected based on age and matching comorbidities. Details of subjects are given in the main text and Tables S1 and S2. The study was approved by the ethics committee of the Medical University of Graz (EK-number: 32–362 ex 19/20).

### Method details

#### Autopsy procedure & specimen collection

Autopsies were performed according to CDC guidelines (https://www.cdc.gov/coronavirus/2019-ncov/hcp/guidance-postmortem-specimens.html) and the epidemic response plan of the county of Styria in a BSL-3 facility that has been specifically designed for post-mortem examinations and sample collection (Loibner et al., 2021). Full autopsies were performed and swabs (eSwab, Copan), tissue and body fluid samples were taken. To omit cross-contaminations between the respiratory tract and the GI tract, the autopsy was sequentially performed. First, the thorax was opened with sterile instruments and lungs and the upper respiratory tract were dissected and sampled. Subsequently, the remaining organs were sectioned with new sterile instruments and sampled. Tissues were immediately fixed in 10% buffered formalin (for histology) or 2,5% buffered (sodium cacodylate; pH 6.5; Sigma) glutaraldehyde (for electron microscopy), snap frozen in liquid nitrogen or preserved in RNAlater (ThermoFisher) and stored at -80°C until further processing.

#### Histopathology and immunohistochemistry

Formalin-fixed paraffin-embedded (FFPE) tissue specimens were processed and stained according to standard procedures. Stains consisted of hematoxylin & eosin (H&E), periodic acid–Schiff (PAS), chromotrope aniline blue (CAB), sirius red, Gomori, Prussian blue, Giemsa and toluidine blue. Chronic renal changes involving the individual renal compartments were scored according to Sethi et al. (Sethi et al. (2017). Liver fibrosis was scored according to Ishak et al. (Ishak et al. (1995). The following antibodies were used: Anti-SARS-CoV-2 nucleoprotein (NP) antibody (dilution 1:100; detection-system: Dako REAL TM EnVision); CD68 (dilution 1:100; detection-system: Ventana UltraView DAB); TTF1 (dilution 1:200; detection-system: Dako K5007); TGFβ1 (dilution 1:50; detection-system: Ventana UltraView DAB); LAG3 (dilution 1:5000; detection-system: Dako K5007); C1q (dilution 1:5000; detection-system: Dako K5007); CD163 (dilution 1:50; detection-system: Ventana UltraView DAB). RNA in-situ hybridization for EBV was performed with the Inform EBER Epstein Barr Virus early RNA kit and the detection-system ISH iView Blue Plus (Ventana).

#### Scoring of histological lung features

From each case multiple specimens from each lobe were taken to account for variations in disease representation (at least 2 specimens per lobe corresponding to 10 specimens per case at least, mean: 13, range: 10-21) and assessed for histopathological features. The histologic progression of DAD includes classically 3 phases (exudative, proliferative, and fibrotic) that correlate with disease duration (Castro, 2006). In our series fibrotic (late) changes were only sparsely present (see Figure 2C) and early and late features of DAD were heterogeneously distributed and often intermixed, thus, we summarized features into (a) early (exudative) and (b) late (proliferative, fibrotic) phases for simplification, as well as included (c) additional features present in DAD histopathology (Hughes and Beasley, 2017). Features consisted of: Interstitial edema/thickening of the alveolo-capillary membranes (non-fibrotic); alveolar edema; hyaline membranes; intra-alveolar (loose) fibrin; scaled of pneumocytes; intra-alveolar proliferation of pneumocytes; alveolar septal fibrosis (early stage organizing DAD); alveolar septal fibrosis (end stage organizing DAD); bronchialisation; squamous metaplasia; virus induced cellular changes in pneumocytes and/or alveolar macrophages; alveolar hemorrhage; interstitial hemorrhage; interstitial hemosidern (free); alveolar hemosiderin (siderophages); fibrin thrombi (capillaries); fibrin thrombi (larger vessels); vascular remodeling (increased density, vascular fissures); intra-alveolar macrophage accumulation; lymphocytes (within alveolo-capillary membranes); lymphocytes (within interstitial space); intra-alveolar (fibro-) cellular infiltrate; lymphocytes (alveolar); megakaryocytes (capillaries); alveolar neutrophils; bronchial neutrophils; a 4-grade scoring system was used to describe the severity of the different pathological features. Score 0 (feature absent), score 1 (feature present in ≤33%), score 2 (feature present in ≤66%), score 3 (feature present in >66%). Scores per slides were summed up and a final score (mean value) was calculated for the respective case.

#### Microbial culture and identification

Native lung tissues were transferred into mixing vessels (ProbeAX; AxonLab) containing 5 mL of physiological saline and were homogenized using a dispersion instrument (ULTRA-TURRAX® Tube Drive; AxonLab). The homogenates were inoculated (0.1 mL aliquots) onto aerobic blood agar, MacConkey agar, chocolate agar, and anaerobic blood agar plates (BD Diagnostics) and into thioglycollate broth (Oxoid). Plates were incubated at 35°C and 37 °C aerobically, in an atmosphere containing 5% carbon dioxide and anaerobically (Genbox anaer, bioMérieux) for up to 14 days, respectively. Cloudy thioglycollate broths were sub-cultured onto plates. Colonies were identified using matrix-assisted-laser-desorption-ionization time-of-flight mass-spectrometry (MALDI-TOF MS) using the Vitek® MS (bioMérieux) or MALDI Biotyper^TM^ (Bruker) instruments or by 16S rRNA gene sequencing (Gorkiewicz et al., 2003).

#### Virus isolation

Lung tissues and swabs from lung parenchyma were used for cultivation of SARS-CoV-2 (Table S3). After mechanical disruption samples were frozen (−80 °C) and thawed (37 °C) twice to increase cell lysis and viral release. 2 mL OptiPro SFM medium (Gibco) with 4 mM L-Glutamine (Gibco) and 1% penicillin-streptomycin (10,000 U/mL; Gibco) were added to the samples. After centrifugation (10 min, 1500 rcf) the supernatants were filtered through a 0.45 μm membrane filter (Millipore) and inoculated on Vero CCL-81 cells with OptiPro SFM medium with 4 mM L-Glutamine and 1% penicillin-streptomycin in T25 flasks (ThermoFisher). After 3–4 days incubation at 37 °C and 5% CO2, the whole cells were mechanically detached with cell scrapers and passaged including the supernatant on to new Vero CCL-81 cells growing in T75 flasks (ThermoFisher). After 1 week the cells were harvested and supernatants were stored after centrifugation (10 min, 1500 rcf) at −80 °C. Viral load was determined by qRT-PCR as described below.

#### RNA extraction

Samples consisted of swabs (eSwab, Copan), tissues and body fluids, the latter were collected with sterile syringes. Fresh tissues were sampled directly into Magna Lyser Green Beads tubes (Roche) pre-filled with 400μL lysis buffer. Tissues were homogenized with a MagnaLyser instrument (Roche) with 6500 rpm for 30 sec. and 3 repetitions. RNA was extracted from 200 μL eSwab solution, 200 μL liquid sample or tissue homogenate using the Maxwell 16 LEV simplyRNA Blood Kit (Promega) according to the manufacturer’s instructions. RNAs from Vero cell cultures were isolated by using the QIAamp Viral RNA Mini Kit (Qiagen) without addition of carrier RNA and transcribed into cDNA with the High-Capacity cDNA Reverse Transcription Kit with RNase Inhibitor (Applied Biosystems) according to manufacturer’s instructions.

#### SARS-CoV-2 quantitative RT-PCR

qRT-PCR for detection and quantification of SARS-CoV-2 in autopsy samples were performed as described (Corman et al., 2020). Briefly, primers, probes and 5 μL of RNA were added to 10 μL of SuperScript III One-Step RT-PCR System with Platinum Taq High Fidelity DNA Polymerase mastermix (ThermoFisher). PCR was performed on a Quantstudio 7 instrument (ThermoFisher) with the following cycling conditions: 55 °C for 15 min, 95 °C for 3 min; 45 cycles consisting of 95 °C for 15 sec and 58 °C for 30 sec. Amplification data was downloaded and processed using the qpcR package of the R project (https://www.r-project.org/). Amplification efficiency plots were visually inspected and Cp2D (cycle peak of second derivative) values were calculated for samples with valid amplification curves. Plots were generated with R using the reshape, tidyverse and ggplot packages. qRT-PCR of virus cultures employed primer sets recommended by the CDC detecting three different regions of the viral nucleocapsid and human RNAseP or GAPDH as control (https://www.cdc.gov/coronavirus/2019-ncov/lab/rt-pcr-panel-primer-probes.html). PCR was performed with the SYBR Green PCR Mastermix (Applied Biosystems) on a Quantstudio 7 instrument (ThermoFisher) with the following cycling conditions: 25 °C for 2 min, 50 °C for 15 min, 95 °C for 10 min, 45 cycles consisting of 95 °C for 3 sec and 55 °C for 30 sec.

#### Viral genome sequencing

PCR primers spanning the whole genome of SARS-CoV-2 were designed yielding in about 2kb amplicons (Table S9). 2.5 μL of RNA were used in three separate RT-PCR reactions as described above with oligonucleotide primers at 400 nM concentration with the following cycling conditions: 55 °C for 15 min, 95 °C for 3 min; 35 cycles consisting of 95 °C for 15 sec and 57 °C for 3 min; final extension at 72 °C for 10 min. PCR products were combined and purified by incubation with 1.8X Ampure XP beads (Beckman Coulter) followed by two washes with 75% ethanol and elution in 30 μL water. Amplicons were fragmented to 150–250 bp length and Ion Torrent barcode and sequencing adapters were ligated to the fragments using the NEBNext Fast DNA Fragmentation & Library Prep Set for Ion Torren kit (New England Biolabs) according to the manufacturer’s recommendations. Libraries were sequenced on an Ion Torrent S5XL instrument using a 540 Chip Kit and the 200 bp sequencing kit (ThermoFisher). Sequences were aligned to the SARS-CoV-2 reference genome (acc. no.:NC_045512.2) using TMAP (v5.10.11) and variants were called with the Torrent Variant Caller (v5.10–12). All called variants were visually inspected and consensus sequences of the viral genomes were generated with bcftools (v1.3.1). Consensus sequences were aligned using clustalw (v2.1) (Larkin et al., 2007), guide trees were visualized in figtree (v1.4.4) and final adjustments were made with Incscape (v0.92). SARS-CoV-2 genomes from our study were uploaded and analyzed with the GISAID SARS-CoV-2 (hCoV-19) databasewhich can be accessed via https://www.gisaid.org/epiflu-applications/next-hcov-19-app/ (Hadfield et al., 2018; Sagulenko et al., 2018).

#### RNA sequencing

Libraries for RNA sequencing (RNA-seq) from lung tissues (19 covid-19 cases #2-#20 and 7 control cases #21, #23-#28) were prepped with the KAPA RNA HyperPrep Kit with RiboErase (HMR) for Illumina® platforms (KAPABIOSYSTEMS) according to the manufacturers protocol. Slight modifications from the protocol consisted of a fragmentation step at 65 °C for 1min, 12 cycles of PCR, as well as an additional bead cleanup at the end of the prep. Libraries were pooled in two pools of 13 samples each by concentration measured with Qubit (ThermoFisher), followed by a bead-cleanup step and an additional QC with Qubit (ThermoFisher) and BioAnalyzer (Agilent). Sequences were resolved on a NovaSeq 6000 Sequencer (Illumina) with a standard paired-end protocol. RNA-seq data were aligned to the human reference genome using STAR (Dobin et al., 2013) (GRCh38 assembly, Ensembl V99 gene models) in 2-pass mode with the following parameters: -sjdbOverhang 100-outFilterMultimapNmax 20-outFilterMismatchNoverLmax 0.05-outFilterScoreMin 0-outFilterScoreMinOverLread 0-outSJfilterReads Unique-outSJfilterOverhangMin 20 15 15 15 15-outSJfilterCountUniqueMin 3 3 3 3-outSJfilterCountTotalMin 3 3 3 3-outSJfilterDistToOtherSJmin 0 0 0 0--outSJfilterIntronMaxVsReadN 100000-alignIntronMin 20-alignIntronMax 100000--alignMatesGapMax 100000-alignSJoverhangMin 12-alignSJstitchMismatchNmax 5–1 5 5-alignSJDBoverhangMin 7-alignSplicedMateMapLmin 0-alignSplicedMateMapLminOverLmate 0.5-limitSjdbInsertNsj 5000000-clip3pAdapterMMp 0.5-outSAMmultNmax 1-outSAMmapqUnique 60-outFilterType BySJout-outSAMunmapped Within-outWigType bedGraph-outReadsUnmapped None SortedByCoordinate-outSAMattrIHstart 1-twopassMode Basic-chimSegmentMin 8-chimOutType Junctions WithinBAM SoftClip-chimScoreMin 1-chimScoreDropMax 20-chimJunctionOverhangMin 8-chimSegmentReadGapMax 3-quantMode GeneCounts-outSAMstrandField intronMotif-outFilterIntronStrands None-chimMainSegmentMultNmax 2-outSAMattributes NH HI AS nM NM MD jM jI XS ch. Alignment to the virus genome reference NC_045512.2 was performed using bowtie2-2.4.1 (Langmead and Salzberg, 2012) on all reads that did not map to the human genome. Read counts on plus-/minus-strand were counted using custom python scripts. Exact positioning of the reads on plus-/minus-strand was done splitting the bam files aligned to NC_045512.2 using samtools-f 0 × 10 and samtools-F 0 × 10 (v0.1.19–44428cd) and bedtools genomecov-ibam BAM NC_045512.2-d (bedtools v2.17.0).

#### RNA profiling

Gene counts were determined using HTSeq (v0.12.4) (Anders et al., 2015) and normalized as fragments per kilobase per million (FPKM) after TMM correction. Gene set variation analysis (GSVA) was performed against a set of immune signatures with xCell (Aran et al., 2017) and means were calculated per cell type using custom R-scripts. Graphs and analyses were generated using R (v.3.6.0). Differential gene expression was conducted using edgeR (Robinson et al., 2010). Differentially expressed genes were selected with FDR<0.05, logCPM>1, and FPKM>1 in at least 5 samples. Clustering of differentially expressed genes was performed using hclust hierachical clustering and subsequent cutting of the gene tree at R function cutree with h = 0.25. Gene set enrichment analysis for clusters was done using the online tool (https://www.gsea-msigdb.org/gsea/msigdb/annotate.jsp) (Subramanian et al., 2005) for canonical pathways and FDR<0.05.

#### Single cell transcriptomic metanalysis

Selected genes from single-cell transcriptomic metadata from Xu et al. (Xu et al. (2020) and Delorey et al. (Delorey et al. (2021) were analyzed with the single-cell atlas database SCovid (Qi et al., 2022).

#### Microbiome analysis based on RNAseq

Microbiome analysis was performed with the following steps using all reads from STAR alignment not mapping to the human reference: quality filtering using fastx-q 30-p 26-Q33 (v0.0.13) cleaning of the fasta file using seqclean-x86_64-N-M-A, realigning to the human reference using blastn against all databases and removal of all reads with 94% similarity. Remaining reads were annotated using MetaPhlAn2 (v2.6.0) (Segata et al., 2012) and Pathseq (GATK v4.1.0.0) (Kostic et al., 2011) with default settings.

#### Microbiome analysis based on the 16S rRNA gene and internal transcribed spacers (ITS)

Bacterial (16S rRNA gene) and fungal (ITS) microbiome analysis from lung tissue was done from FFPE samples which enabled us to preselect samples based on histology showing unambiguous pathology (i.e. DAD vs. pneumonia). DNA was extracted from FFPE tissues using the Maxwell 16 Tissue DNA Purification Kit (Promega). DNA concentration was measured by Picogreen fluorescence. The variable V4 region of the bacterial 16S rRNA gene was amplified with PCR using oligonucleotide primers 16S_515_f and 16S_806_r (Maiwald, 2011) from 50 ng DNA extracted from lung tissue. Likewise, fungal ITS sequences were amplified with primers ITS1 and ITS2 (Halwachs et al., 2017). PCR was performed using the 16S Complete PCR Mastermix Kit (Molzym). The first PCR reaction product was subjected to a second round of PCR with primers fusing the 16S/ITS primer sequence to the A and P adapters necessary for Ion Torrent sequencing whileadditionally including a molecular barcode sequence to allow multiplexing of up to 96 samples simultaneously. PCR products were subjected to agarose gel electrophoresis and the band of the expected length (about 330 bp) was excised from the gel and purified using the QIAQick (Qiagen) gel extraction system. DNA concentration of the final PCR product was measured by Picogreen fluorescence. Amplicons from up to 60 samples were pooled equimolarly and sequencing was performed on Ion Torrent XL benchtop sequencer using the Ion 400 bp sequencing chemistry (all reagents from ThermoFisher). Sequences were split by barcode and transferred to the Torrent suite server. Raw bam-files comprised of single-end reads generated by NGS, were converted from bam files to fastq.gz files by using samtools (Danecek et al., 2021). Quality control and preprocessing of sequences was performed using FastQC (version 0.7), MultiQC (version 1.7) and trimmomatic (version 0.36.5) using following parameters: LEADING:3 TRAILING:3 SLIDINGWINDOW:4:15 MINLEN:200. Sequence processing and microbiome analysis was performed using QIIME2 (version 2020.6) (Bolyen et al., 2019). After quality filtering all samples with less than 9833 reads/sample were excluded from downstream analysis. In concordance with RNA-Seq analysis Covid-1 was excluded for sub-analysis (cause of death: myocardial infarction), resulting in 18 Covid-19 and 12 control samples for 16S analysis (average frequency: 28201.7 reads/sample). For ITS analysis only 1 sample showed more than 9833 reads/per sample (case #18: 10272 reads). All other samples showed no clear ITS signal with a sequencing depth of maximum 219 reads per sample and were therefore discarded. Denoising, dereplication and chimera filtering of single-end reads were performed using DADA2 (denoise-pyro) (Callahan et al., 2016). 16S-based analysis was performed with the latest SILVA 138 taxonomy and the Naive Bayes classifier trained on Silva 138 99% OTUs full-length sequences (Quast et al., 2013). For ITS-based analysis a classifier was trained on the UNITE reference database (ver8-99-classifier; 04.02.2020) (Nilsson et al., 2019) according to John Quensen (http://john-quensen.com/tutorials/training-the-qiime2-classifier-with-unite-its-reference-sequences/; assessed 20/08/2020). Differences in microbial composition between groups were tested with implemented QIIME2 plugins using PERMANOVA (p < 0.05, qiime diversity beta-group-significance: Bray-Curtis, Jaccard, Unweighted UniFrac, Weighted UniFrac) and Kruskal-Wallis (p < 0.05, qiime diversity alpha-group-significance: Observed features, Shannon, Evenness, Faith PD). For metagenomic biomarker discovery taxonomic feature-tables served as input for the LEfSe (linear discriminant analysis effect size) method (Galaxy version 1.0; p < 0.05, LDA>2, All-against-all) (Segata et al., 2011). Plots were generated with R (version 3.6.2)6 in RStudio (1.1.463)7 using following packages: tidyverse (1.3.0)8, qiime2r (0.99.6)9, ggplot2 (3.3.3)10, dplyr (1.0.6)11 and ggpubr (0.4.0.999)12 and GraphPad Prism. The graphical abstract was created with BioRender (www.BioRender.com).

#### Protein isolation and western blot

Proteins from lung tissues were extracted with TRIzol® (ThermoFisher) according to the supplier’s protocol. Briefly, tissue homogenates were subjected to phase separation wherein the organic phase containing the protein was further processed. Four volumes of ice-cold acetone were added to the organic phase and the mixture was incubated at −20 °C overnight, followed by a centrifugation step (13000 rpm) at 4 °C for 15 min. The supernatant was discarded and the pellets were dried at 60 °C for 60 min. Subsequently, 100 μL RIPA buffer (Sigma) containing protease inhibitors and phosphatase (0.1 mM Pefabloc, 1 mM DTT, 1X cOmplete^TM^ Mini, 1X PhosSTOP^TM^) and 1 % SDS (Roche) were added and the mixture incubated at 65 °C for 90 min. Supernatants were transferred to a new Eppendorf tube and 100 μL 8 M urea in 0.05 M Tris (pH 8,5) and 1% SDS were added and incubated at 55 °C for 30 min. Corresponding supernatants and pellets were pooled and transferred to 2 mL MagNA Lyser tubes (Roche) with ceramic beads and homogenized 2 times at 6500 rpm for 20 sec. Samples were incubated on ice for 10 min and subsequently centrifugated with 13000rpmat 4 °C for 15 min. Supernatants were transferred to new Eppendorf tubes. For western blotting proteins were mixed with 4X Laemmli buffer (Bio-Rad) and incubated for 10minat 95 °C and then loaded onto 11% (v/v) SDS-PAGE gels (Amersham) and electrophoresed at 80 mA for 2 h and subsequently transferred onto nitrocellulose membranes (Amersham). Blotting efficiency was determined with Ponceau staining (Ponceau S solution, Sigma). Non-specific binding was blocked with 5% (w/v) non-fat dry milk (Bio-Rad) in TRIS-buffered saline and 0.1% (v/v) Tween 20 (Merck) for 1 h. Subsequently, the membranes were incubated with antibodies against C1q (Dako Denmark A/S 1:5000), TGFß1 (Cell Signaling Technology, 1:1000), and GAPDH (Cell Signaling Technology, 1:1000) overnight at 4 °C. Thereafter, membranes were washed and incubated with the appropriate HRP-conjugated secondary antibody (Amersham, ECL Anti-Rabbit IgG, 1:5000). Immunolabeling was detected using ECL Select Western Blot Reagent (Amersham) and visualized with the ImageQuant™ LAS 500 instrument (Amersham). GAPDH was used as loading control to determine protein abundance and band density was quantified and compared by using ImageJ.

### Quantification and statistical analysis

GraphPad Prism and R were used for data analysis and imaging. All data are represented as means ± SD if not otherwise specified. Statistical significance testing employed the Mann-Whitney and Kruskal-Wallis tests and p-values <0.05 were considered statistically significant. Correlation analyses employed Spearman and Pearson correlation. For differentially gene expression and gene enrichment analyses a False Discovery Rate (FDR) <0.05 was used. Permanova was used for statistical determination in the Principal Component Analysis (PCA). The *n* number is specific for the number of human subjects.

## Data Availability

The RNAseq, 16S rRNA gene and ITS amplicon sequencing data has been deposited in the European Nucleotide Archive (ENA): PRJEB45873. This paper does not report original code. Any additional information required to re-analyze the data reported in this paper is available from the lead contact upon request.

## Supplemental information

Document S1. Figures S1–S18

Table S1. Clinical data of the study cohort, related to Figures 2C–2G and 3C

Table S2. Laboratory parameters of the study cohort, related to Figure 2F

Table S3. Virus isolation, related to Figures 1D and S2

Table S4. Viral genotypes, related to Figures 1E and 1F

Table S5. Viral genome variants, related to Figures 1E and 1F

Table S6. Histopathology of covid-19, related to Figures 2A and S4–S9

Table S7. Microbial culture, related to Figures 3G and S10

Table S8. Dominant microbes, related to Figures 3G and S10

Table S9. Primers for SARS-CoV-2 genome sequencing, related to STAR Methods and Figure 1C
