# Supplementary material for: Host and microbiome features of secondary infections in lethal covid-19
Source: iScience. 2022 Aug 13;25(9):104926. doi: 10.1016/j.isci.2022.104926 (PMC9374491; doi:10.1016/j.isci.2022.104926)
Supplement: Document S1. Figures S1–S18 [file mmc1.pdf]

iScience, Volume *25*

Supplemental information

Host and microbiome features

# of secondary infections in lethal covid-19

Martin Zacharias, Karl Kashofer, Philipp Wurm, Peter Regitnig, Moritz Schütte, Margit Neger, Sandra Ehmann, Leigh M. Marsh, Grazyna Kwapiszewska, Martina Loibner, Anna Birnhuber, Eva Leitner, Andrea Thüringer, Elke Winter, Stefan Sauer, Marion J. Pollheimer, Fotini R. Vagena, Carolin Lackner, Barbara Jelusic, Lesley Ogilvie, Marija Durdevic, Bernd Timmermann, Hans Lehrach, Kurt Zatloukal, and Gregor Gorkiewicz

Supplementary Figures

Figure S1. Clinical courses of covid-19 cases, Related to Figure 2D and 2G.
Figure S2. Relation of SARS-CoV-2 cultivability and transcript levels determined by RNA-seq, Related to Figure 1D.
Figure S3. Gross pathology representation of lungs, Related to Figure 2A.
Figure S4. Histological representation of DAD in covid-19, Related to Figure 2A.
Figure S5. Immunohistochemical analyses of DAD, Related to Figure 2A.
Figure S6. Neutrophils correlation and clinical parameters, Related to Figure 2G.
Figure S7 Kidney histopathology, Related to Figure 1A.
Figure S8. Heart histopathology, Related to Figure 1A.
Figure S9. Liver Histopathology, Related to Figure 1A.
Figure S10. Dominant taxa identified by molecular methods and cultivation in covid-19 lungs, Related to Figure 3F and 3G.
Figure S11. Disease phase and deconvolution subgroup association, Related to Figure 5A.
Figure S12. Correlation analyses of C1q levels with disease duration, Related to Figure 6C.
Figure S13. Single cell transcriptomic analysis of *C1q* and macrophages, Related to Figure 6.
Figure S14. Single cell transcriptomic analysis of *LAIR-1* and macrophages, Related to Figure 7A.
Figure S15. Transcriptomic correlation of *C1q*, *LAIR-1* and *LILRB4* from RNA-seq, Related to Figure 7B.
Figure S16. TGF beta 1 western blots, Related to Figure 7C.
Figure S17. Inhibitory immune checkpoints in covid-19 and controls from RNA-seq, Related to Figure 7D.
Figure S18. Correlation analysis of inhibitory immune checkpoints and cell types derived from RNA-seq, Related to Figure 7D and 7F.

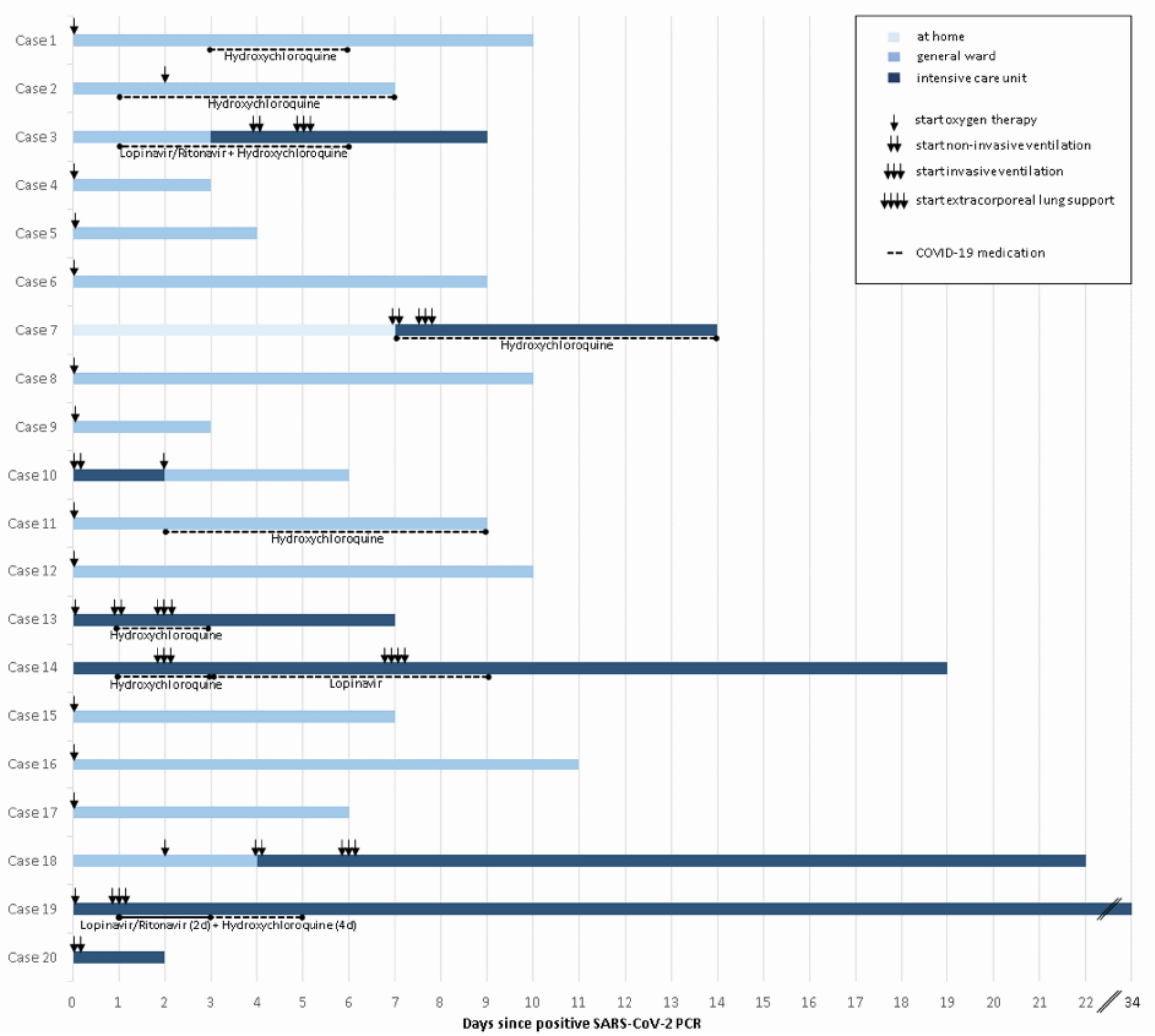

Figure S1. Clinical courses of covid-19 cases, Related to Figure 2D and 2G. X-axis specifies interval from SARS-CoV-2 positive PCR. Treatment in the general ward and/or intensive care unit is shown. Start of ventilation therapies is indicated by arrows. Covid-19 specific therapies are indicated.

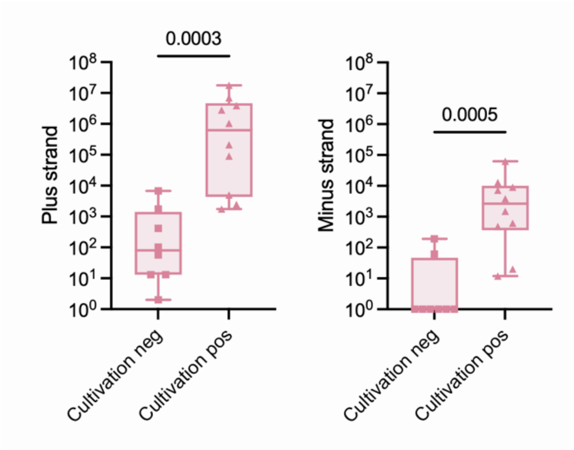

Figure S2. Relation of SARS-CoV-2 cultivability and transcript levels determined by RNA-seq, Related to Figure 1D, (Mann-Whitney test).

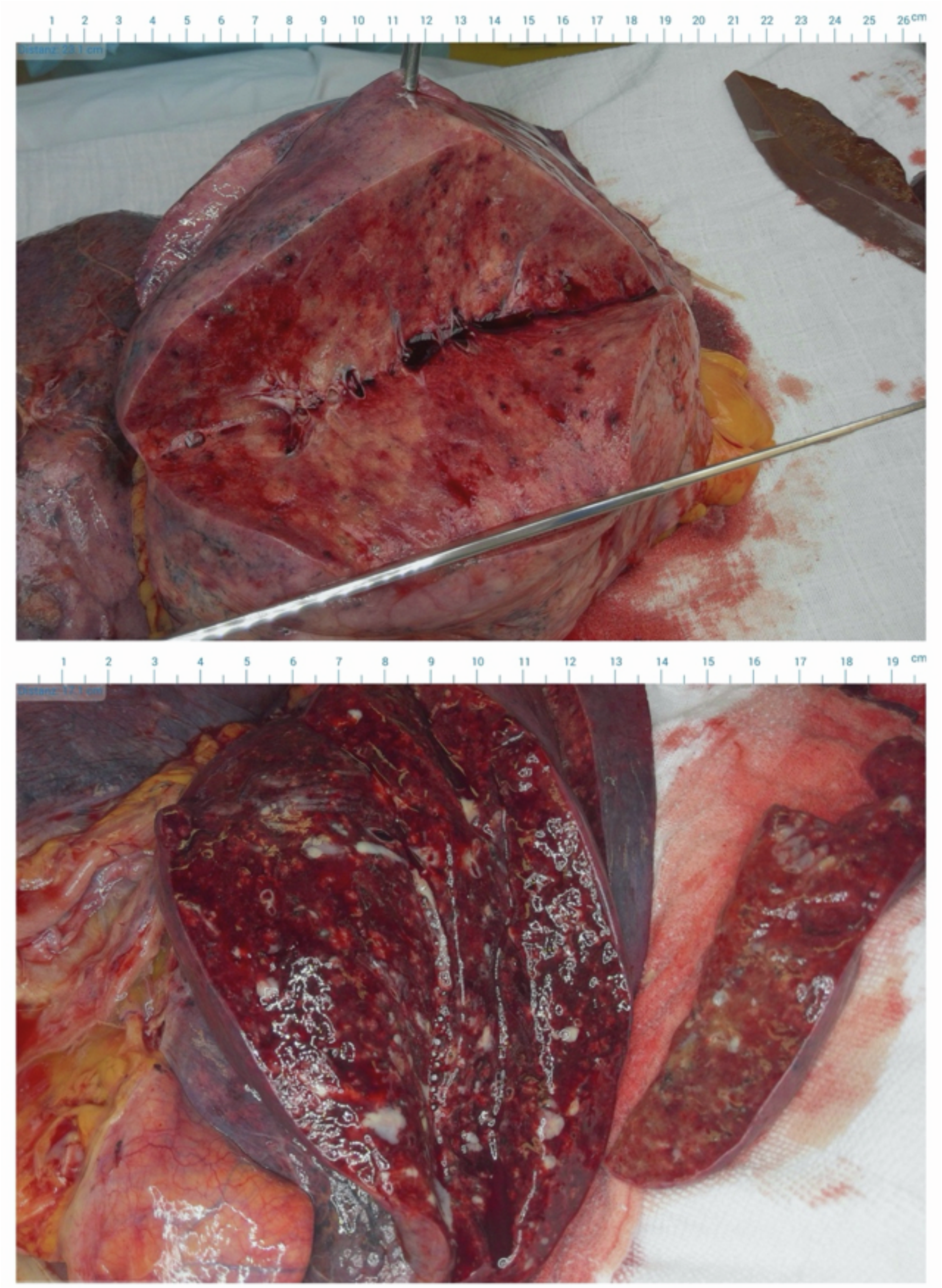

Figure S3. Gross pathology representation of lungs, Related to Figure 2A. (Top) Section through a lobe with DAD. The lung parenchyma is inhomogeneously colored and consolidated. (Bottom) A case with bacterial superinfection shows pus in bronchial lumina and on the cut surface of the lung.

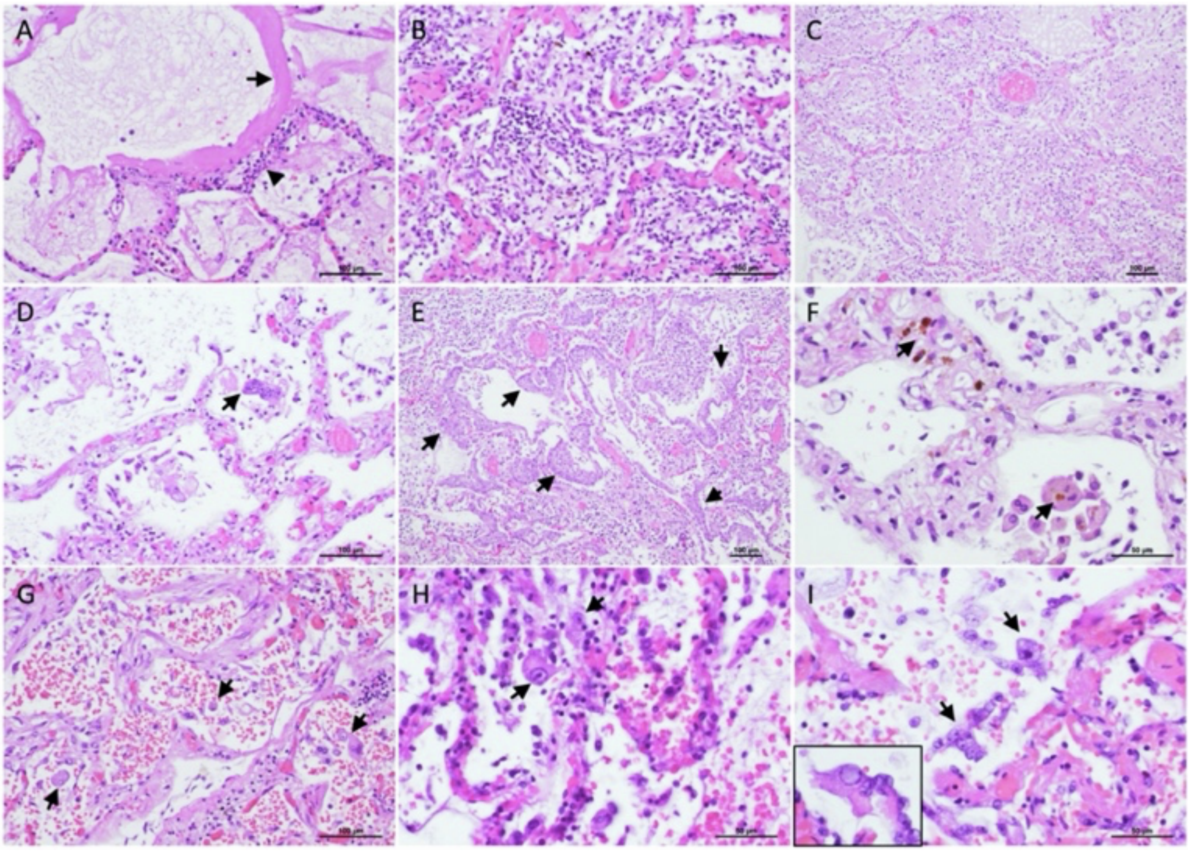

Figure S4. Histological representation of DAD in covid-19, Related to Figure 2A. (A) Exudative DAD with hyaline membranes (arrows) and lymphocytic interstitial infiltrates in alveolar septa (arrowhead). (B, C) Organizing DAD with intra-alveolar fibro-cellular infiltrates. (D) Multinucleated syncytial pneumocytes in organizing DAD (arrow). (E) Squamous metaplasia in organizing DAD (arrows). (F) Hemosiderin in alveolar septa and alveolar macrophages (arrows). (G-I) Pneumocyte nuclear atypia with macro-nucleoli (arrows; "owl-eye" in H). Atypical pneumocytes are often scaled off the alveolar membrane. In G also alveolar hemorrhage is evident. Inset in I shows ground-glass nuclear changes in atypical pneumocytes.

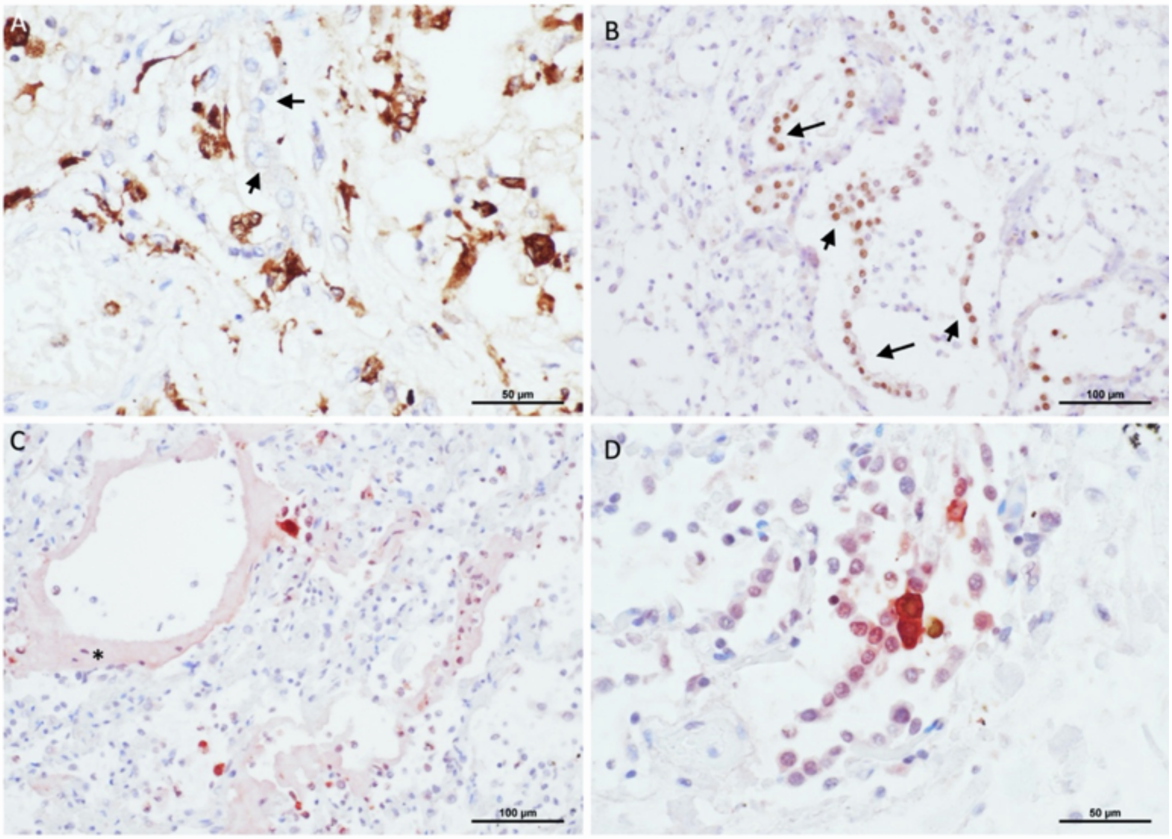

Figure S5. Immunohistochemical analyses of DAD, Related to Figure 2A. (A) Macrophage specific CD68 staining. Note the non-stained atypical pneumocytes (arrows). (B) Pneumocytes are scaled-off from the alveolar membrane (pneumocyte specific nuclear TTF-1 staining, arrows). (C, D) SARS-CoV-2 nucleoprotein staining of infected pneumocytes; asterisk (*) marks a hyaline membrane.

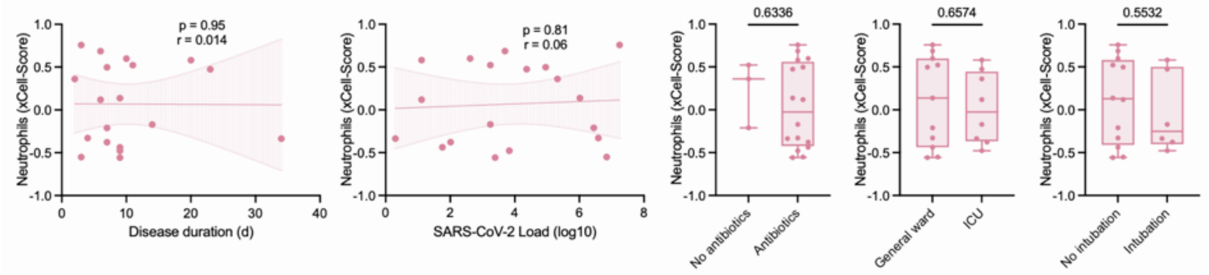

Figure S6. Neutrophils correlation and clinical parameters, Related to Figure 2G. Correlation analyses of neutrophil abundance (determined by deconvolution of RNA-seq data with xCell) and clinical parameters (Spearman correlation, Mann-Whitney test).

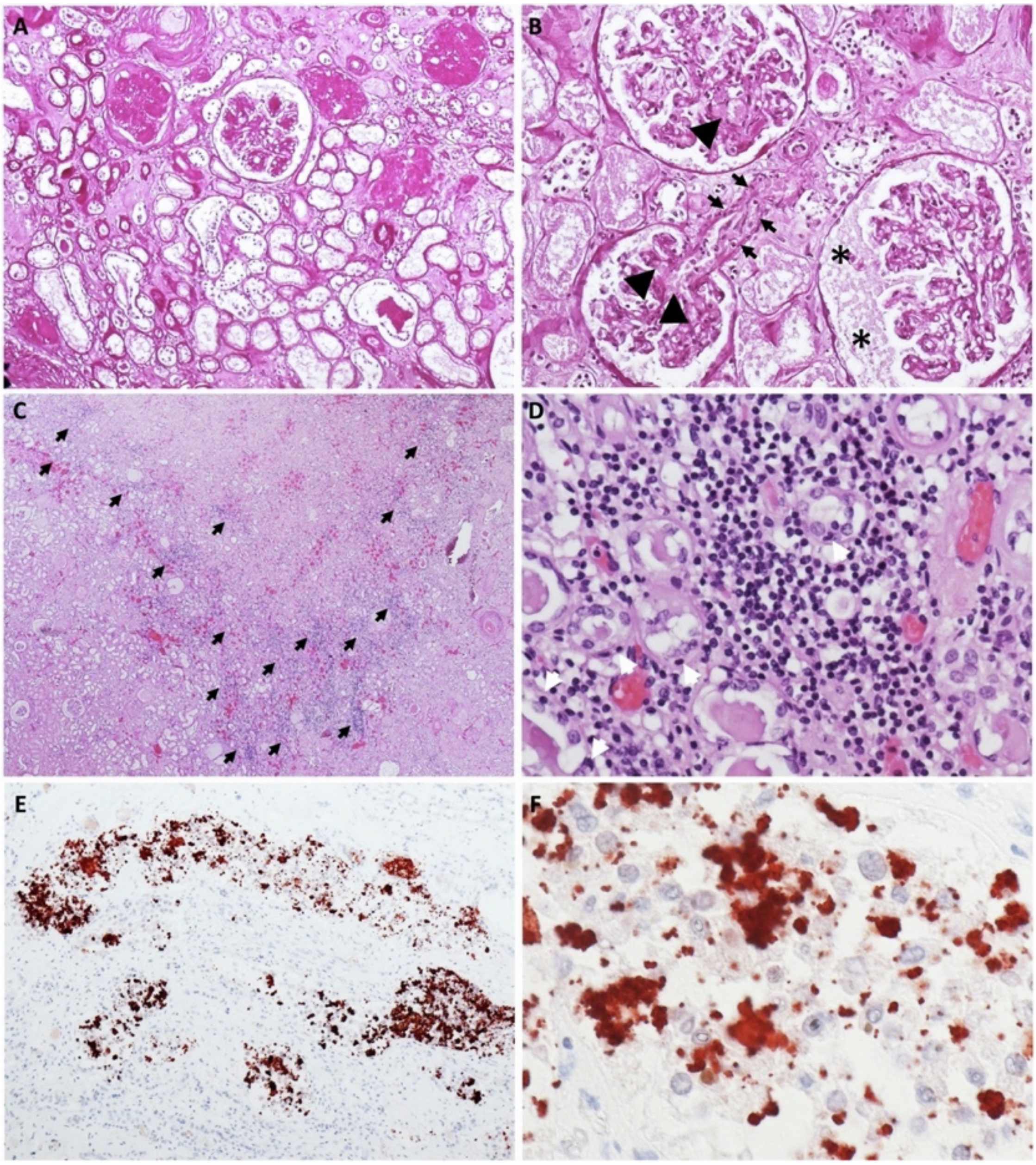

Figure S7 Kidney histopathology, Related to Figure 1A. (A) PAS-stained kidney specimen with advanced diffuse and nodular diabetic glomerulosclerosis, advanced parenchymal atrophy and severe arteriolohyalinosis (100-fold). (B) PAS-stained kidney specimen showing segmental obliteration of glomerular capillary loops by fibrin thrombi (arrowheads), endothelitis of a glomerular arteriole (arrows) and accumulation of plasma in Bowman's space (asterisks; 200-fold). (C) Overview of an H&E-stained kidney specimen with diffuse lymphocytic tubulo-interstitial nephritis (arrows; 20-fold). (D) H&E-stained kidney specimen showing severe tubular epithelial changes: loss of brush border, nuclear swelling and cytoplasmic vacuolisation. Note the tubulo-interstitial inflammatory cell infiltrate, mainly consisting of lymphocytes with foci of tubulitis, as characterized by the presence of mononuclear cells on the basolateral aspect of the tubular epithelial cells (400-fold). (E, F) Immunohistochemical detection of SARS-CoV-2 nucleoprotein showing granular staining predominantly in the cytoplasm and occasionally in the nuclei of tubular epithelial cells of large distal tubules (100-fold & 600-fold, respectively).

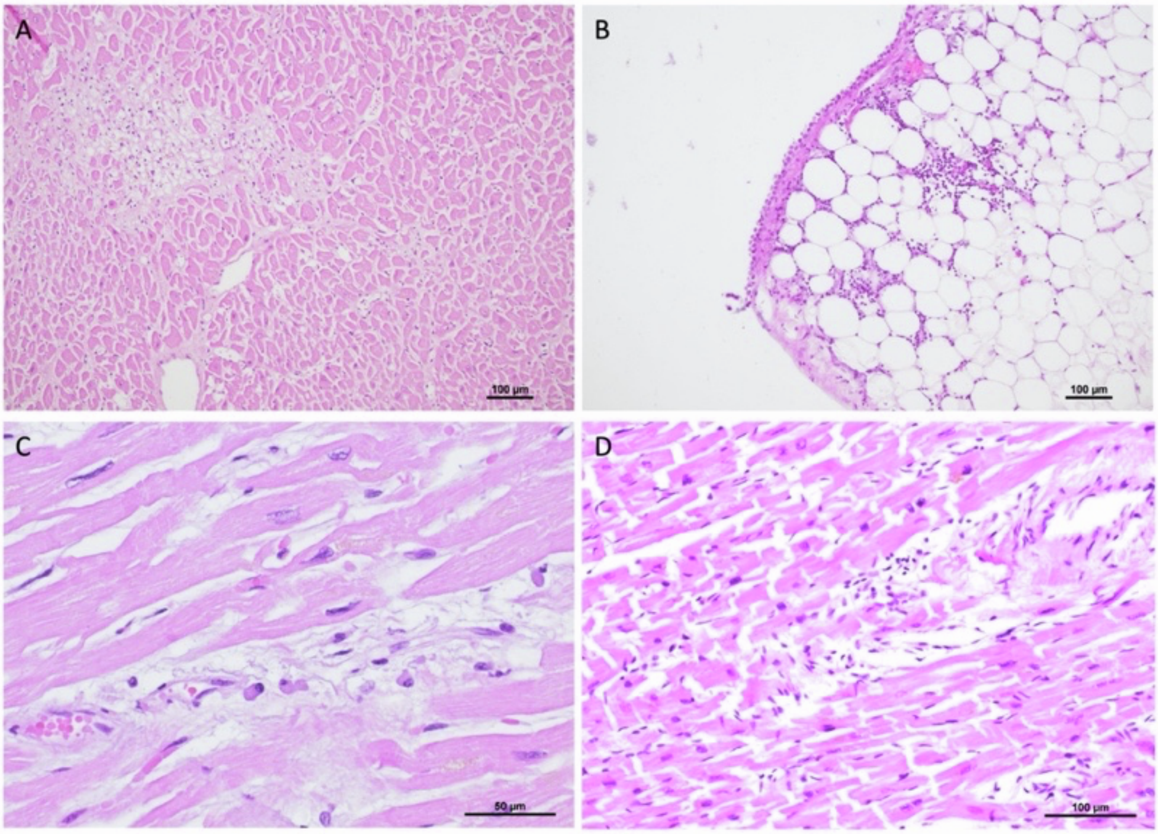

Figure S8. Heart histopathology, Related to Figure 1A. (A) Fresh small scar in the myocardium. (B) Sparse subepicardial lymphocytic infiltrate. (C, D) Single plasma cells and lymphocytes in the endomysium around vessels.

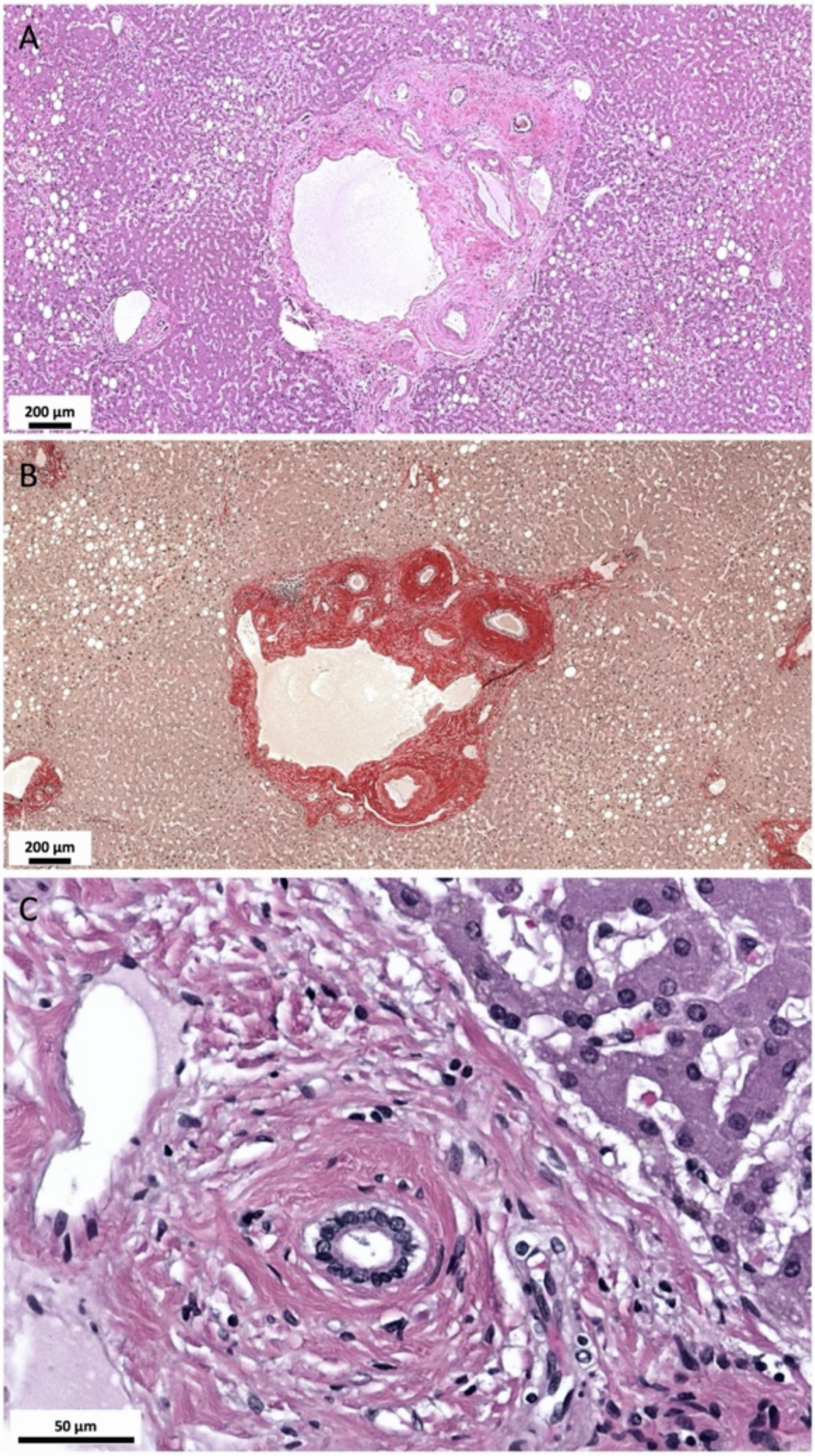

Figure S9. Liver Histopathology, Related to Figure 1A. Morphological changes resembling sclerosing cholangitis COVID 19. **(A)** Preserved lobular architecture. Mild macrovesicular steatosis of the lobular parenchyma. Liver septum with mild lymphocytic infiltrates and septal bile duct adjacent to the A. hepatica branch surrounded by thick layer of condensed collagen fibers (50-fold; hematoxylin & eosin). **(B)** Sclerosis is highlighted by the sirius red connective tissue stain (50-fold; sirius red). **(C)** Portal tract with interlobular bile duct and periductal sclerosis.

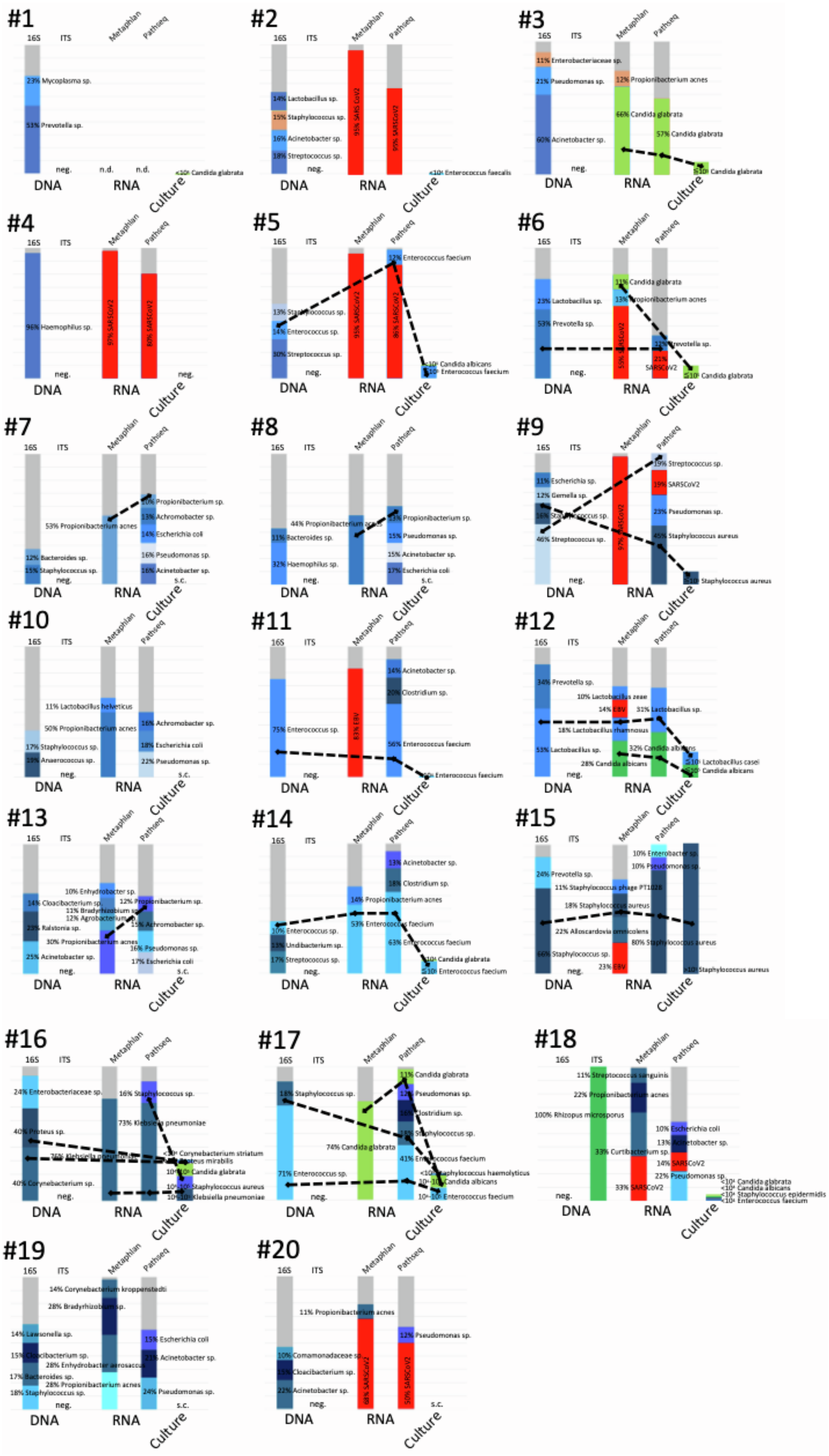

Figure S10. Dominant taxa identified by molecular methods and cultivation in covid-19 lungs, Related to Figure 3F and 3G. Shown is the relative abundance of 16S rRNA gene and ITS sequencing and microbial RNA-seq data annotated with Metaphlan or Pathseq pipelines.

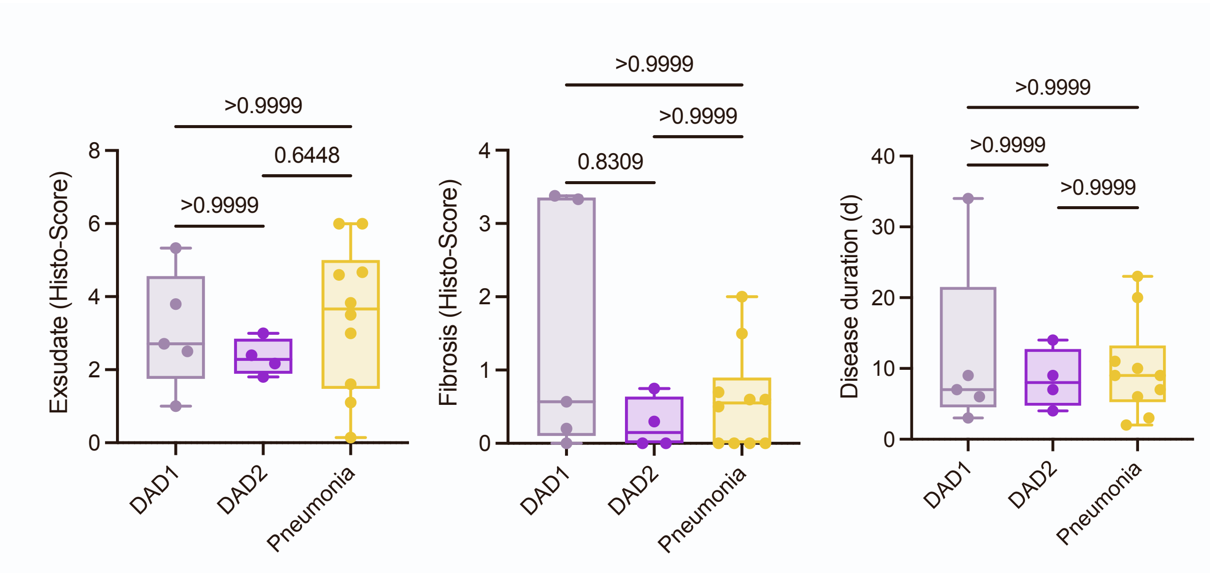

Figure S11. Disease phase and deconvolution subgroup association, Related to Figure 5A. No statistically significant difference between early and late DAD features as well as disease duration with deconvolution groups (Kruskal-Wallis).

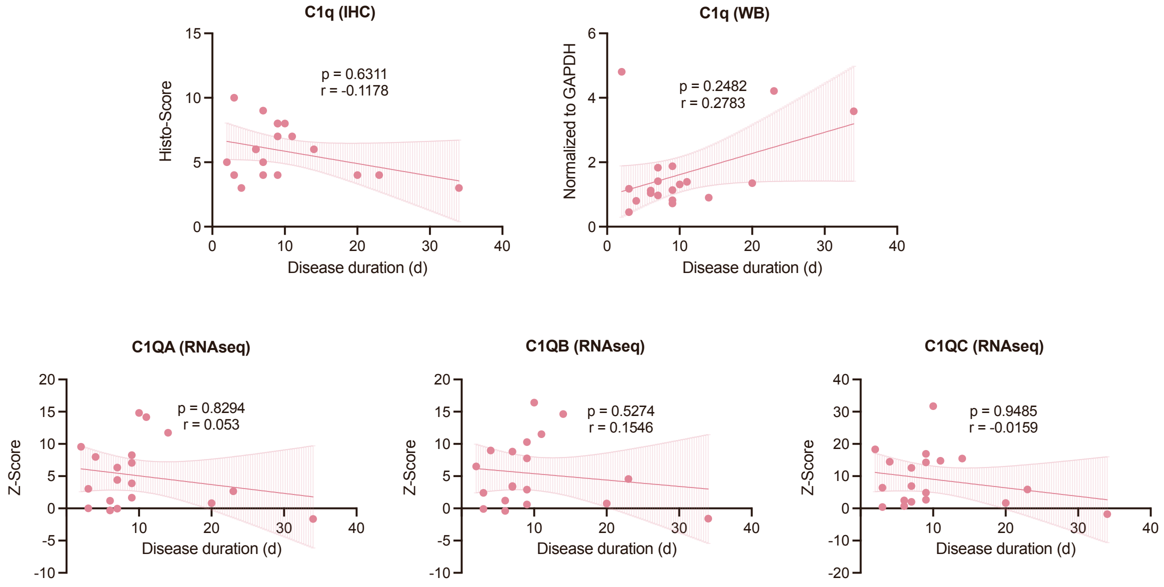

Figure S12. Correlation analyses of C1q levels with disease duration, Related to Figure 6C. No significant correlation with C1q levels based on IHC, western blot or RNA-seq with disease duration (Spearman r).

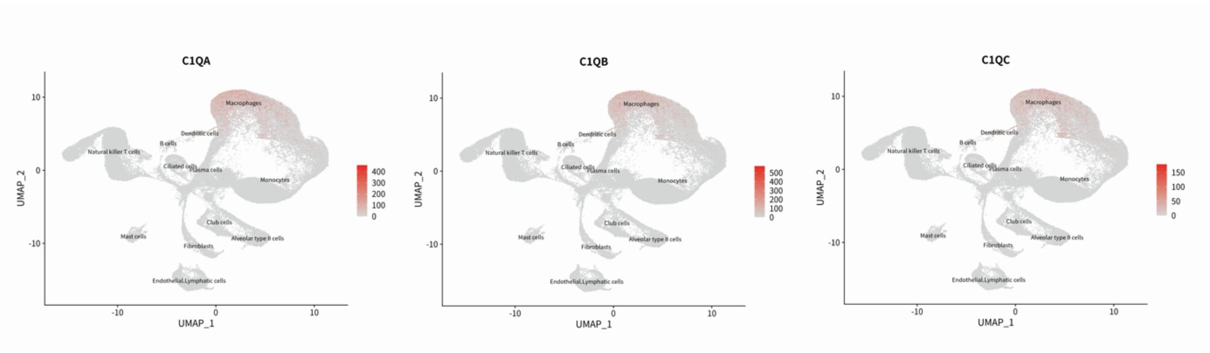

Figure S13. Single cell transcriptomic analysis of *C1q* and macrophages, Related to Figure 6. *C1q A, B* and *C* chains are mainly expressed by macrophages. Data derived from Xu et al (Xu et al., 2020) and analyzed with SCovid single-cell atlas database (Qi et al., 2022).

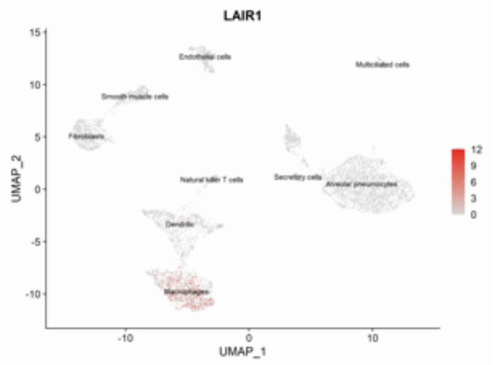

Figure S14. Single cell transcriptomic analysis of *LAIR-1* and macrophages, Related to Figure 7A. *LAIR-1* is mainly expressed by macrophages. Data derived from Delorey et al (Delorey et al., 2021) and analyzed with SCovid single-cell atlas database (Qi *et al.*, 2022).

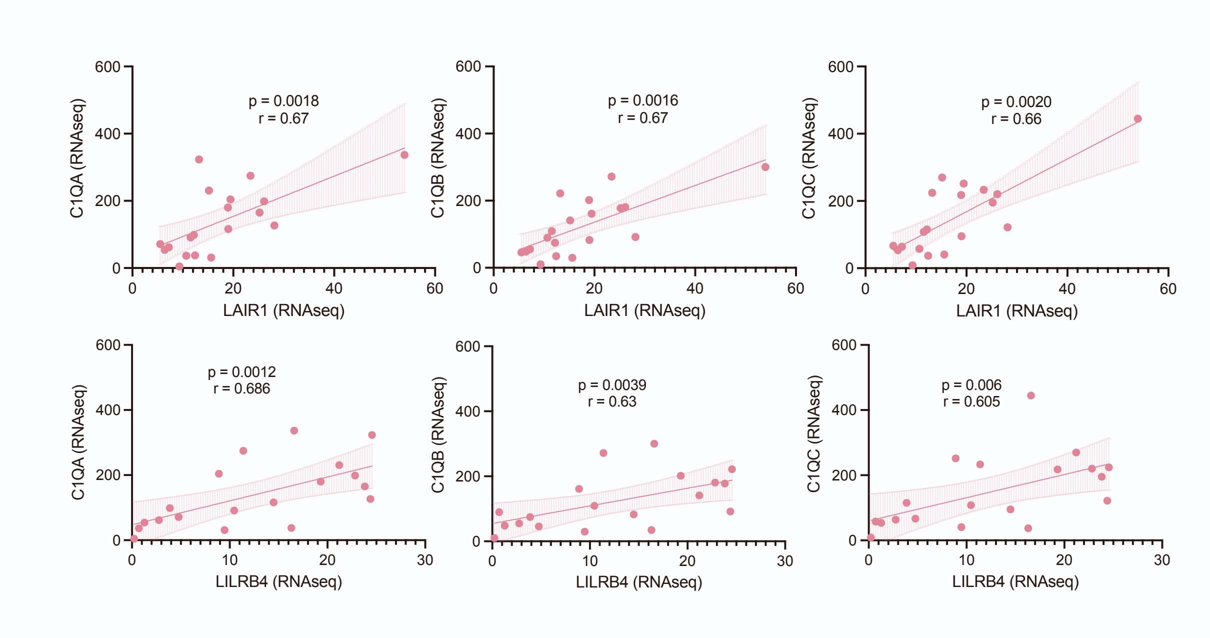

Figure S15. Transcriptomic correlation of *C1q*, *LAIR-1* and *LILRB4* from RNA-seq, Related to Figure 7B. Correlation of *C1q* chains with *LAIR-1* (top) and *LILRB4* (bottom; Spearman r).

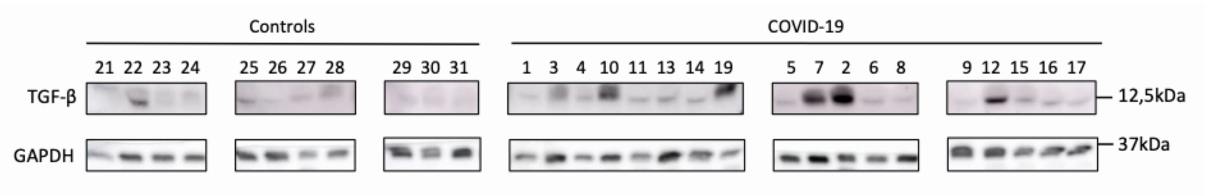

Figure S16. TGF beta 1 western blots, Related to Figure 7C. TGF beta 1 (12.5 kDa) protein in covid-19 lung tissues and controls (reference human GAPDH).

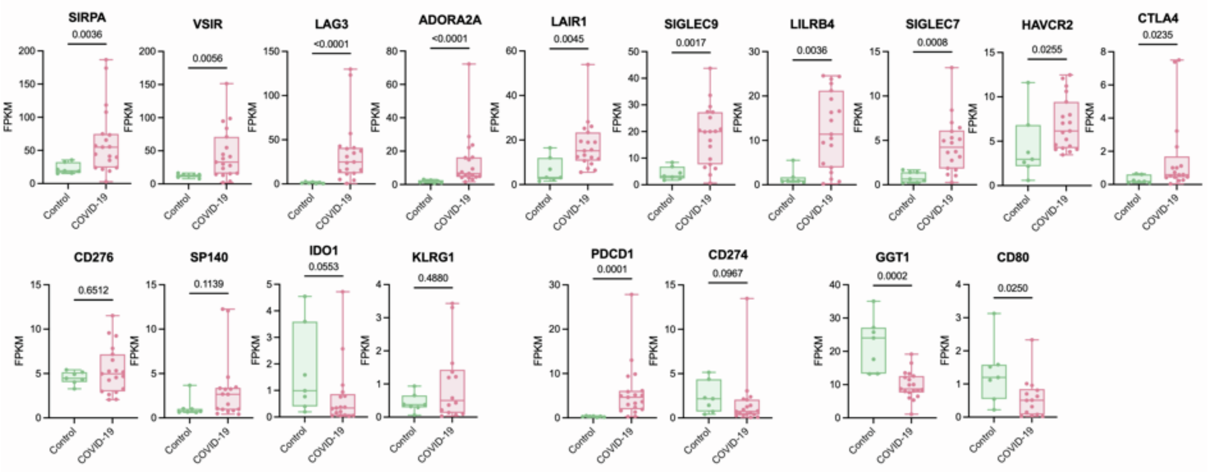

Figure S17. Inhibitory immune checkpoints in covid-19 and controls from RNA-seq, Related to Figure 7D. Immune checkpoint inhibitor expression in covid-19 compared to controls (Mann-Whitney test).

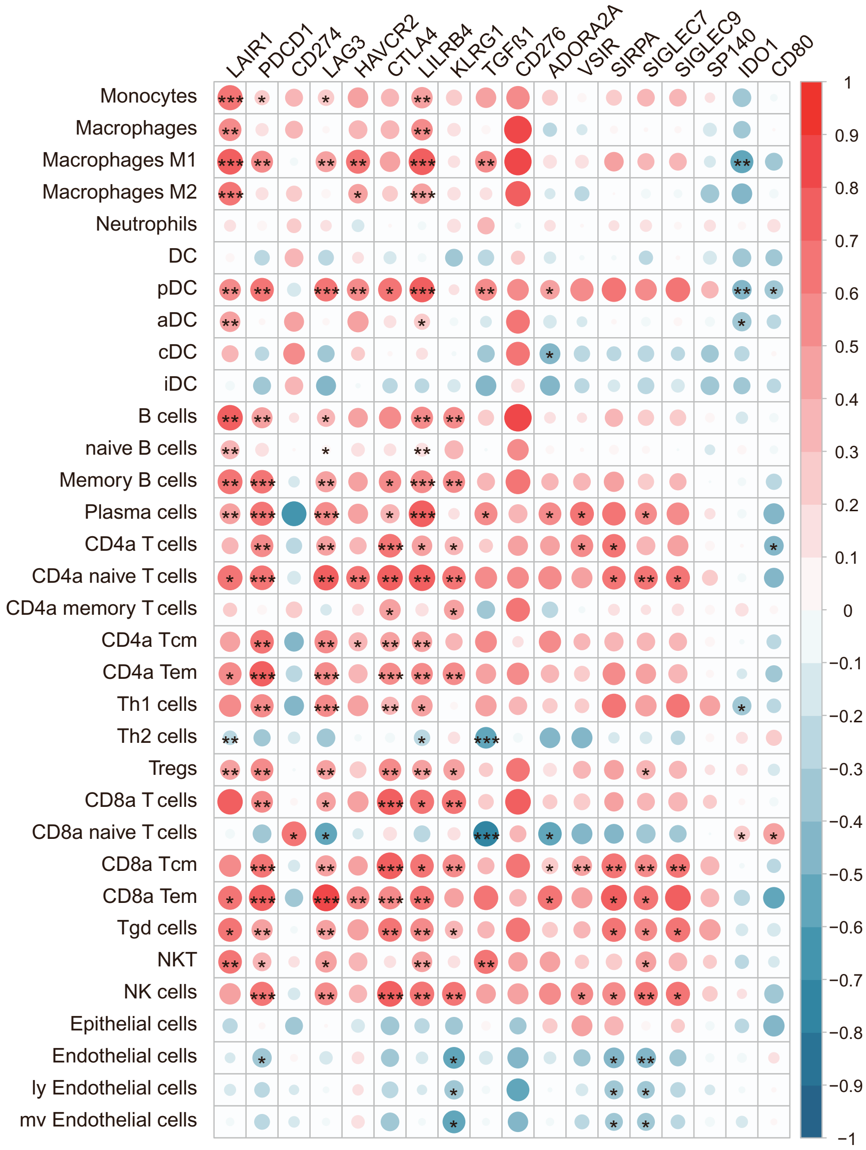

Figure S18. Correlation analysis of inhibitory immune checkpoints and cell types derived from RNA-seq, Related to Figure 7D and 7F. Cell types derived from xCell analysis (Spearman correlation; p*<0.05, p***<0.001).
